# Spatial transcriptome analysis of the tea tender shoot sheds light on transcriptional regulation of characteristic metabolites

**DOI:** 10.1093/hr/uhag003

**Published:** 2026-01-06

**Authors:** Cheng Zhang, Chengzhe Zhou, Caiyun Tian, Shengjing Wen, Zhendong Zhang, Anru Zheng, Zhenhan Rui, Yuting Li, Shuaibo Shao, Siwei Deng, Zhong Wang, Yuqiong Guo

**Affiliations:** Anxi College of Tea Science, College of Horticulture, Fujian Agriculture and Forestry University, Fuzhou 350002, China; Anxi College of Tea Science, College of Horticulture, Fujian Agriculture and Forestry University, Fuzhou 350002, China; Fujian Collaborative Innovation Center for Green Cultivation and Processing of Tea Tree in Universities, Fujian Agriculture and Forestry University, Anxi County, Quanzhou 362400, China; Anxi College of Tea Science, College of Horticulture, Fujian Agriculture and Forestry University, Fuzhou 350002, China; Anxi College of Tea Science, College of Horticulture, Fujian Agriculture and Forestry University, Fuzhou 350002, China; Anxi College of Tea Science, College of Horticulture, Fujian Agriculture and Forestry University, Fuzhou 350002, China; Anxi College of Tea Science, College of Horticulture, Fujian Agriculture and Forestry University, Fuzhou 350002, China; Anxi College of Tea Science, College of Horticulture, Fujian Agriculture and Forestry University, Fuzhou 350002, China; Anxi College of Tea Science, College of Horticulture, Fujian Agriculture and Forestry University, Fuzhou 350002, China; Anxi College of Tea Science, College of Horticulture, Fujian Agriculture and Forestry University, Fuzhou 350002, China; Anxi College of Tea Science, College of Horticulture, Fujian Agriculture and Forestry University, Fuzhou 350002, China; Anxi College of Tea Science, College of Horticulture, Fujian Agriculture and Forestry University, Fuzhou 350002, China; Fujian Collaborative Innovation Center for Green Cultivation and Processing of Tea Tree in Universities, Fujian Agriculture and Forestry University, Anxi County, Quanzhou 362400, China; Anxi College of Tea Science, College of Horticulture, Fujian Agriculture and Forestry University, Fuzhou 350002, China; Fujian Collaborative Innovation Center for Green Cultivation and Processing of Tea Tree in Universities, Fujian Agriculture and Forestry University, Anxi County, Quanzhou 362400, China

## Abstract

The tender shoots of tea plant [*Camellia sinensis* (L.) Kuntze] contain characteristic flavor metabolites such as catechins, caffeine, and theanine, which are the raw materials for making various types of high-quality tea. The gene expression profiles with spatial information for tea shoots remain unclear, which has hindered the exploration of precise regulatory mechanisms of these characteristic metabolites in different cell types. Here, we provided a high-throughput analysis of the spatial gene expression of the tea shoot, including the apical bud, young leaf, and stem. The genome-wide expression pattern was delineated into nine representative spatial coexpression clusters, and cell type identification was achieved by integrating histological structures with marker gene annotation. The dynamic differentiation processes of cells in leaf and bud were revealed through the reconstruction of pseudotemporal trajectories, uncovering the coupling relationship between spatial organization and developmental progression. Gene Ontology enrichment analysis indicated that different clusters were enriched in functional pathways such as photosynthesis, cell wall construction, substance transport, and hormone response during differentiation, demonstrating their stage-specific expression throughout development. Additionally, we found that structural genes associated with the metabolism of catechins, theanine, and caffeine exhibited distinct spatial expression patterns across various tissues. Based on functional verification, we identified that the transcription factor gene *CsTCP4* could positively regulate the biosynthesis of catechins and the hydrolysis of theanine. In conclusion, the spatial transcriptome atlas provides a foundational dataset for understanding gene expression heterogeneity in tea shoots and expands our understanding of the synergistic regulation of theanine and catechin metabolism in tea.

## Introduction

The tender shoots of the tea plant [*Camellia sinensis* (L.) Kuntze], including tender stems, leaves, and buds, are the raw materials for making various types of high-quality tea [1, 2]. After water, tea ranks second worldwide in beverage consumption, consumed by two-thirds of the world’s population [3]. The appeal of tea stems from the presence of various distinctive secondary metabolites such as catechins, theanine, and caffeine, all of which are beneficial to human health and play a significant role in determining the pleasant flavor quality [4–6]. Among these, the phenol-ammonia ratio is a key indicator determining the processing suitability of tea. It refers to the ratio of polyphenols (primarily catechins) to free amino acids (primarily theanine), reflects the balance between bitter taste and freshness of tea [7]. Therefore, targeted regulation of the relative levels of catechins and theanine in tea leaves is of great significance for determining the final flavor quality of the finished tea product. Recent progress in genomics and the study of plant metabolism has greatly advanced our knowledge, enabling a clearer elucidation of both the biosynthetic pathways and foundational regulatory mechanisms of these metabolites in the tea plant [8]. The metabolism of flavor metabolites underwent stringent multilevel regulation, especially at the transcriptional level [9]. For example, the CIN-type CsTCP3/4 transcription factors (TFs) interact with MBW complex (CsTT8/CsMYB75) to form heterodimer, which activates the promoter of catechin synthesis gene *CsANS1*/*CsANR1* [10]. CsMYB40 and CsHHO3 TFs bind to the theanine synthesis gene *CsAlaDC* promoter, dynamically modulating theanine biosynthesis through opposing regulatory modes in response to nitrogen availability [11]. These studies have partially elucidated the regulatory mechanisms of catechins and theanine metabolism, but information regarding the specific cell types involved in these processes within tea shoots is lacking, and it remains unknown whether any regulatory factors can coordinately modulate these two major classes of metabolites. To date, the primary method for identifying key genes involved in the synthesis of secondary metabolites in tea plants has been RNA sequencing (RNA-seq) technology, which utilizes mixed tissue samples to screen for differentially expressed genes. However, this approach can only provide averaged gene expression profiles across cell populations, leading to the loss of low-abundance information. Consequently, the gene expression characteristics of specific cell types may be masked, and the heterogeneity of gene expression among cells cannot be adequately revealed. Elucidating the spatial distribution of gene expression within cells of tea shoots is crucial for deciphering the precise regulatory mechanisms governing key characteristic metabolites.

With the development of single-cell RNA sequencing (scRNA-seq) technology, researchers can reveal specific gene expression patterns at the single-cell level, which is helpful to analyze cell heterogeneity and identify cell types [12]. In recent years, a number of studies have utilized scRNA-seq to investigate issues related to tissue development and the regulation of secondary metabolism in tea plants. Wang *et al*. [13] reported the single-cell level atlas of first and third tea leaves and identified a glycosyltransferase gene exclusively expressed in mesophyll cells, functionally linked to catechin ester modification. A cell type-specific transcriptional landscape of tea plant roots, generated via scRNA-seq, uncovered a multicellular compartmentalization strategy for theanine biosynthesis [14]. Moreover, through single-nucleus RNA sequencing (snRNA-seq) of tea root tips, a high-resolution atlas containing 37 922 nuclei was constructed, identifying eight major cell types and their specific marker genes (such as the lateral root cap-specific gene *CsLAX1*), revealing the cell type-specific distribution of key genes involved in the synthesis of catechins, theanine, and caffeine [15]. In addition, Zhao *et al*. [16] systematically characterized the cell type composition and metabolic features during bud-to-leaf development in tea plants by integrating snRNA-seq, bulk RNA-seq, and metabolomic analyses. They found that catechin biosynthetic enzymes were predominantly localized in palisade mesophyll cells, while theanine accumulated mainly during the bud stage. These studies indicated that analyzing gene expression patterns at the single-cell level helped identify novel genes that regulate critical secondary metabolites in tea plants. However, the application of scRNA-seq is limited by its inherent loss of spatial information from tissue dissociation. Additional constraints in nonmodel species and thick-walled tissues include the inefficiency of protoplast extraction in some differentiated cell types and the current dependency on known marker genes for spatial mapping [17]. Understanding the physical interactions between cells, vital for tissue development and physiological function, requires knowledge of their spatial location. The need to correlate gene expression with cellular position is fundamental and has driven the creation of spatial transcriptome (ST) technologies. In 2017, a pioneering study by Giacomello *et al*. [18] demonstrated the feasibility of ST in plants by first optimizing its key steps, including tissue fixation, staining, and permeabilization. This demonstration was achieved by successfully generating ST maps for the *Arabidopsis thaliana* inflorescence meristem, as well as for developing and dormant leaf buds of *Populus tremula* and female cones of *Picea abies*. With the continuous improvement of ST sequencing technology, the ST information of many horticultural plant tissues such as tomato (*Solanum lycopersicum*) [19] and peach (*Amygdalus persica*) [20], has been elucidated.

In the present study, we constructed the spatially resolved transcriptomics analysis of the tea tender shoot based on 10 × Visium technology. The specific marker genes for each cluster and cell type of tea leaves were identified. According to precise spatiotemporal transcripts, we delineate developmental trajectories of tea tender leaf cells and infer gene expression signatures associated with cell fate decisions. Moreover, we identified CsTCP4 through ST analysis, revealing that it is involved in the coregulation of the metabolism of catechins and theanine. Overall, we provide an example of the application of ST analysis in tea plants and reveal insights into the complex developmental regulation of catechins, theanine, and caffeine metabolisms.

## Results

### Identification of the structure and cell type of tender stem, leaf, and bud of the tea shoot

**Figure 1:**
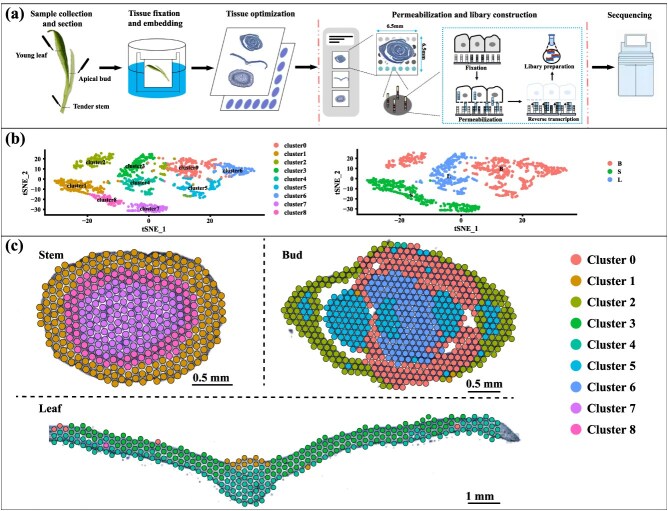
Spatially resolved transcriptome analysis of tender stem, leaf, and bud of tea shoot. (a) A workflow for sampling and ST sequencing of tender stem, leaf, and bud of tea shoot. (b) t-SNE representation of the nine unsupervised clusters. (c) Spatial distribution of different clusters.

To acquire high-quality ST profiles, tender shoots of *C. sinensis* var. *sinensis* cv. ‘Tieguanyin’ were selected as experimental materials. Fresh tissues (stem, young leaf, and apical bud) were rapidly frozen using an isopentane–liquid nitrogen bath and embedded in optimum cutting temperature (OCT) compound (Fig. 1a). Given the dimensions of ST slides (6.5 × 6.5 mm^2^) and the small size of tea shoot tissues, individual samples could not fully cover the capture area; thus, 3–5 tissue sections were mounted per slide. Transverse sections were subsequently prepared, with structurally intact tissues selected for histological observation and downstream data analysis (Figs S1 and S2). To evaluate the stability and reproducibility of ST data in the absence of multiple biological replicates, three structurally intact and anatomically comparable regions were selected from each tissue type (stem, leaf, and bud) for whole-transcriptome correlation analysis. The calculated Pearson correlation coefficients revealed exceptionally high intratissue consistency (*R*^2^ > 0.98; Fig. S3), demonstrating the robustness and representativeness of the transcriptomic data across spatially independent regions. These findings confirm that the ST dataset provides a reliable foundation for subsequent biological interpretation. Structurally intact single-tissue sections were then selected for histological observation and downstream transcriptomic analysis. The stem, functioning as an axial structure connecting roots to leaves, flowers, and fruits, provides mechanical support, enables conduction, and participates in storage of nutrients and moisture. Immature stems with leaves are designated shoots. In transverse sections, tender stems exhibited an oval shape, featuring a single-layered epidermis covered by a thin cuticle. Beneath the epidermis, 3–4 layers of collenchyma cells were observed, delivering mechanical support particularly crucial for young plants. Continuous parenchyma tissues surrounded the vascular system, where xylem formed a ring encircling the pith, and phloem was positioned externally to the xylem. The pith occupied nearly half the stem volume, comprising large, round-to-ovoid parenchymatous cells. Leaves, as the primary harvest organs for tea production and key sites for photosynthesis, displayed three components in sections: epidermis, mesophyll, and veins. The epidermis enveloping the entire leaf comprised adaxial epidermis on the ventral surface and abaxial epidermis on the dorsal surface. Mesophyll between adaxial epidermis and abaxial epidermis consisted of palisade mesophyll and spongy mesophyll. The palisade mesophyll adjacent to adaxial epidermis contained 1–3 layers of tightly packed cylindrical cells perpendicular to the epidermis, while the spongy mesophyll beneath it exhibited loosely arranged, irregular rounded cells with prominent intercellular spaces. Veins forming a reticulate network throughout the mesophyll served conductive and supportive roles, with vascular bundles derived from procambium constituting their core; vascular bundles contained xylem toward the adaxial side and phloem toward the abaxial side, complemented by parenchyma cells below the phloem. Bud sections shared cellular similarities with leaves, though young leaves in the central bud displayed only abaxial epidermis, adaxial epidermis, and parenchyma cells without observable apical meristems. Collectively, the stem, leaf, and bud sections demonstrated well-preserved histological integrity, rendering them suitable for comprehensive ST sequencing.

### Spatiotemporal gene expression in tender stem, leaf, and bud of tea plant

To provide ST atlases of different tissues in tea plants for comprehensive understanding of their complexity, tissue sections were subjected to RNA extraction and quality control. The results showed RIN values exceeding 7 in all samples, indicating no RNA degradation during tissue freezing and suitability for subsequent experiments (Table S1). The ST slides contained 5000 barcoded spots with a diameter of 55 μm. Each spot captured ~1–10 cells and included unique molecular identifiers (UMIs). Intact tissue sections were mounted onto slides, and ST sequencing was performed on the 10× Visium platform. Among 199–217 million reads per sample, 87%–94% mapped to the ‘Tieguanyin’ reference genome. The proportions of reads mapped to intronic versus exonic regions were 3.3%, 2.7%, and 3.4% versus 61%, 72%, and 68% in stem, leaf, and bud tissues, respectively. Space Ranger removed duplicate UMIs per barcode, visualized tissue capture areas and spatial barcode locations, and distinguished reads per spot. Statistics for reads per spot, median genes detected, and UMIs (representing gene expression per spot) are summarized in Table S2. Structurally intact stem, leaf, and bud tissues underwent dimensionality reduction via principal component analysis (PCA), were clustered into nine distinct clusters, and were simultaneously visualized based on t-Distributed Stochastic Neighbor Embedding (t-SNE) (Fig. 1b). The left panel displayed nine gene clusters (Clusters 0–8) via unsupervised clustering, while the right panel categorized clusters by tissue origin. Clusters showed clear separation in 2D coordinates, indicating high intracluster homogeneity and significant intercluster divergence (e.g., spatial segregation between adjacent Clusters 1 and 2 along the tSNE_2 axis; Clusters 7 and 8 at peripheral locations suggested unique expression signatures). Bud spanned broad regions, reflecting transcriptional diversity and cross-cluster distribution. Leaf tissues distributed centrally to leftward in band-like patterns, adjacent to stems and overlapping partially with buds, indicating transitional or bridging functions. Stem clustered compactly in the tSNE_1-negative region, suggesting functional specialization.

Marker genes for each cluster were identified based on cluster-specific expression patterns, providing clues for tissue regionalization and functional annotation (Table S3). Three clusters (Clusters 1, 7, 8) were observed in stems; five clusters (Clusters 0, 1, 3, 4, 8) in leaves; and six clusters (Clusters 0, 1, 2, 4, 5, 6) in buds (Fig. 1c). Each cluster corresponded to specific cell types: Cluster 0 (phloem, spongy mesophyll, and vascular bundle in buds); Cluster 1 (epidermis, cuticle, collenchyma, and parenchyma in stems); Cluster 2 (xylem, phloem, vascular bundle, and spongy mesophyll in buds); Cluster 3 (vascular bundle and spongy mesophyll in leaves); Cluster 4 (xylem, phloem, spongy mesophyll, and parenchyma in leaves); Cluster 5 (xylem, phloem, and vascular bundle in buds); Cluster 6 (spongy mesophyll, xylem, and phloem in buds); Cluster 7 (pith in stems); Cluster 8 (xylem and phloem in stems).

Cluster annotation was validated using *A. thaliana* single-cell transcriptomic data from PlantCellMarker (Table S3), and marker genes exhibited high concordance with anatomical origins. In stems, *CsTGY01G0001198* (Plant invertase/pectin methylesterase inhibitor superfamily protein) and *CsTGY12G0001085* (lipid-transfer protein, LTP) were highly expressed in Cluster 1, marking the epidermal and cortical regions, whereas *CsTGY02G0003271* (sucrose synthase 4, SUS4) in Cluster 7 served as a pith marker reflecting its role in carbohydrate storage. In leaves, *CsTGY01G0003171* (RBCS1B) and *CsTGY06G0000745* (RBCS1A) were specifically expressed in photosynthetically active mesophyll cells within Cluster 3, while *CsTGY07G0001788* (xyloglucan endotransglucosylase/hydrolase, XTH) and *CsTGY12G0001774* (LTP) were enriched in Cluster 4, corresponding to xylem and phloem regions involved in substance transport and cell wall remodeling. In buds, *CsTGY14G0002498* (carbonic anhydrase) and *CsTGY02G0003271* (SUS4) marked metabolically active vascular and mesophyll tissues within Cluster 0, while *CsTGY06G0001309* (YABBY transcription factor) and *CsTGY02G0000881* (thaumatin-like protein) were enriched in Cluster 5, suggesting roles in developmental regulation and defense responses. Overall, stem-related clusters were mainly associated with defense, carbohydrate storage, and vascular transport; leaf clusters were linked to photosynthesis and nutrient translocation; and bud clusters were involved in vascular specialization, cell wall modification, and developmental plasticity. These findings highlight the organ-specific functional partitioning revealed by the spatial transcriptomic atlas.

To further enhance the credibility of cross-species annotation, we integrated tea-specific tissue marker genes previously validated in independent studies and mapped them onto our spatial transcriptomic dataset via BLAST alignment [13, 21] (Table S4). The majority of these tea-specific markers exhibited spatial expression patterns highly consistent with their known tissue identities. For instance, the leaf xylem-specific gene *CsTGY06G0000427* showed strong expression in Cluster 4 (leaf xylem-enriched region); the stem phloem marker *CsTGY08G0000936* was highly expressed in Cluster 8 (stem vascular bundle); and the leaf epidermis marker *CsTGY03G0001244* displayed high expression in Cluster 3, consistent with its roles in epidermal defense and gas exchange. Interestingly, certain tissue-specific genes also exhibited transcriptional activity in noncorresponding tissues—e.g., the leaf phloem-specific gene *CsTGY11G0000423* was expressed not only in leaf Clusters 3 and 4 but also prominently in bud Cluster 0, suggesting transcriptional continuity of vascular systems during early bud–leaf developmental transitions. Based on the spatial transcriptomic data, three genes—*CsTGY06G0000015* (encoding LTP*)*, *CsTGY01G0003171* (encoding RBCS1B*)*, and *CsTGY09G0000131* (encoding desiccation-related protein)—were selected as potential tissue-specific marker genes. Further examination of their expression profiles (FPKM values) across different tissues in the ‘Tieguanyin’ cultivar revealed that *CsTGY06G0000015* was predominantly expressed in stem tissue, *CsTGY01G0003171* in leaf tissue, and *CsTGY09G0000131* in bud tissue, with minimal or negligible expression in other organs [22]. Accordingly, the corresponding high-expression tissues were selected as experimental targets for RNA fluorescence *in situ* hybridization (FISH) to validate their spatial expression patterns (Table S5 and Fig. S4). The FISH results showed that the fluorescence signal of *CsTGY06G0000015* was primarily localized in the phloem and xylem regions of the stem. Although the fluorescence signals of *CsTGY01G0003171* and *CsTGY09G0000131* were unevenly distributed within their respective tissues, *CsTGY01G0003171* was mainly localized to mesophyll cells in the leaf, while *CsTGY09G0000131* was enriched in the palisade and spongy mesophyll regions of the bud. These spatial expression patterns were consistent with those observed in the spatial transcriptomic analysis, thereby validating the accuracy and reliability of spatial transcriptomics in resolving tissue-level gene expression landscapes. Taken together, the spatial transcriptomic atlas accurately captures the molecular and functional compartmentalization of tea shoots and provides a robust spatial framework for dissecting tissue specialization and developmental dynamics in woody plants.

### Development trajectories of tea tree shoots and leaf cells

**Figure 2:**
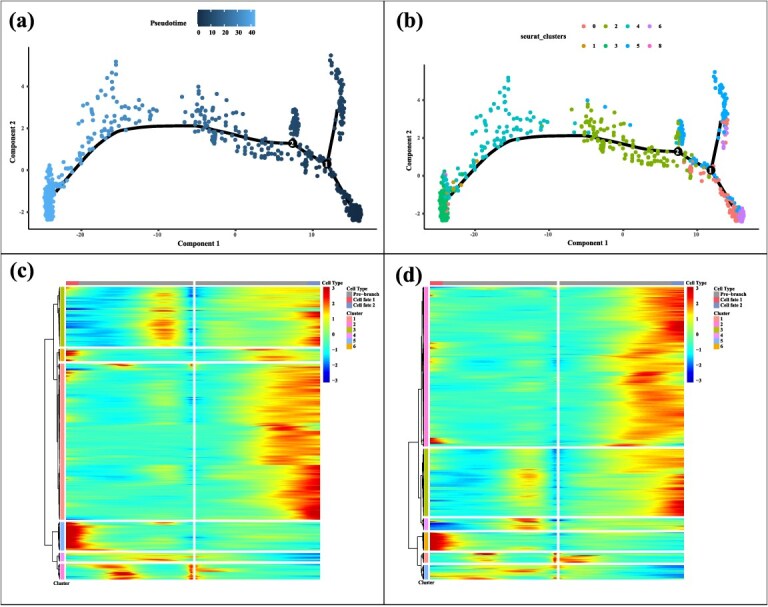
Developmental trajectories of tea bud and leaf. (a) Pseudotime trajectory distribution plot. (b) Pseudotime trajectory plot of cell clusters. This panel shows the trajectories of different cell clusters as they differentiate over time, each dot represents a cell. (c) Heatmap of gene expression dynamics along Branch 1 of the pseudotime trajectory. Genes are clustered into six modules based on expression patterns across pseudotime. The top annotation bar indicates cell fate classification (prebranch, fate 1, fate 2), and the side color bar represents gene clusters. (d) Heatmap of gene expression dynamics along Branch 2 of the pseudotime trajectory. Similar to (c), genes are grouped into six clusters, reflecting distinct temporal expression programs along the branch toward defense- or support-related cell fates.

Pseudotime trajectory reconstruction was performed for bud and leaf tissues, which exhibited higher developmental plasticity compared with relatively stable stem cells. The trajectory revealed a continuous transition from early undifferentiated states to functionally specialized cells, including photosynthetic and vascular lineages (Fig. 2a and b).

To systematically dissect this process, we integrated pseudotime trajectory reconstruction with Gene Ontology (GO) enrichment analysis (Fig. S5, Table S6) to investigate the functional transitions across different clusters. Early-stage Clusters 0 and 6, mainly derived from bud tissues, were characterized by stress response, hormone signaling, and chloroplast assembly, reflecting unspecialized cells with strong environmental adaptability. Intermediate clusters (2, 4, 5) displayed signatures of cell wall remodeling, transmembrane transport, and vascular differentiation, suggesting transitional states during bud-to-leaf development. Late-stage Clusters 1 and 3 were enriched in ribosome biogenesis, photosynthesis, and metabolic pathways, corresponding to mature photosynthetic and vessel-like cells in leaf tissues. Notably, Cluster 4 also showed enrichment in intercellular connectivity and reproductive development, implying additional specialization in signaling and reproductive functions.

To gain deeper insights into the dynamic regulation of cell fate within tea shoot tissues, we systematically compared the two major branches (Branch 1 and Branch 2) derived from the pseudotime trajectory. By integrating trajectory-based clustering with gene expression heatmaps and functional enrichment analyses, we uncovered marked differences between the two branches in terms of temporal progression and biological specialization (Fig. 2c and d, Table S7). Branch 1 followed a canonical developmental route from undifferentiated cells toward photosynthetically and metabolically active cell types. Clusters 1 and 2 were enriched in membrane components, oxidoreductase activity, and hormone signaling pathways, indicative of early-stage cellular structuring and environmental responsiveness. Clusters 3 and 4 were associated with transcriptional regulation and organelle biogenesis, representing intermediate differentiation stages. Clusters 5 and 6 showed strong enrichment for photosynthesis, amino acid biosynthesis, and energy metabolism pathways, suggesting terminal differentiation into metabolically specialized mesophyll or vascular cells. This trajectory aligns well with the classical model of leaf development, wherein precursor cells progressively acquire mature physiological functions. In contrast, Branch 2 represented a distinct developmental trajectory with a terminal fate skewed toward defense-related or supportive cell identities. Clusters 1 and 2 were enriched in GO terms such as ‘chitin catabolic process’ and ‘cell wall macromolecule catabolic process’, reflecting active cell wall remodeling and structural reorganization. Clusters 3 and 4 highlighted membrane proteins, energy metabolism, and signaling pathways, suggesting a state of activation or lineage commitment. Notably, Clusters 5 and 6 were strongly enriched in pathways related to stress response, protein processing, and plant–pathogen interactions, indicating a differentiation route toward immune-responsive or structurally supportive cell types, potentially representing specialized subpopulations within the bud or vascular system.

Overall, the pseudotime trajectory analysis not only revealed a continuous functional transition during tea shoot development, but also illustrated the spatiotemporal coordination of cell fate diversification. The two branches respectively culminate in metabolically specialized and defense-oriented cell identities, offering a novel spatial transcriptomic perspective on the coordinated mechanisms between tissue development and metabolic function in tea.

### Spatial expression of genes related to the metabolism of catechins in tea shoot

**Figure 3:**
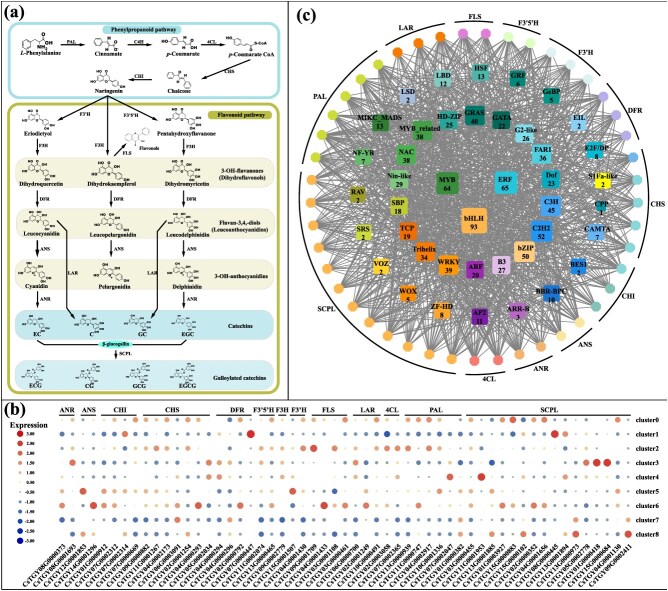
Spatial expression information of relevant structural genes in tea catechin biosynthesis and potential transcriptional regulation networks in tea tender shoot. (a) The biosynthesis pathway of catechins in tea plants. *ANR*, *ANS*, *CHI*, *CHS*, *DFR*, *F3′5′H*, *F3H*, *F3′H*, *FLS*, *LAR*, *4CL*, *PAL*, and *SCPL* represent genes encoding *anthocyanidin reductase*, *anthocyanidin synthase*, *chalcone isomerase*, *chalcone synthase*, *flavonoid 3′,5′-hydroxylase*, *flavanone 3-hydroxylase*, *flavanone 3′-hydroxylase*, *flavonol synthase*, *leucoanthocyanidin reductase*, *4-coumarate-CoA ligase*, *phenylalanine ammonia-lyase*, and *serine carboxypeptidase-like acyltransferase*, respectively. (b) Heatmap shows the spatial expression pattern of catechin biosynthesis pathway genes in tea plants. (c) Interaction network between structural genes and potential TFs in the catechin biosynthesis pathway. Based on the coexpression patterns of genes across different groups, TFs potentially involved in regulation were screened by identifying those that exhibited expression trends consistent with the structural genes. The colored octagons represent the structural genes associated with catechin biosynthesis. The color-coded squares represent TFs potentially regulating structural genes, with gray lines depicting regulatory connections between these TFs and structural genes. The numerical annotations indicate the gene count of the corresponding TFs.

Catechins belong to flavonoids, play a predominant role in determining the characteristic bitter and astringent flavor profile of tea [23]. These components consist of several structurally distinct molecules, including non-ester type catechins (C, EC, EGC, and GC), which are further acylated to form ester-type catechins (designated as CG, ECG, EGCG, and GCG) by *serine carboxypeptidase-like* (*SCPL*) [24] (Fig. 3a). The biosynthesis of catechins related to flavonoid metabolism, related genes have been identified [25]. There are *chalcone synthase* (*CHS*), *chalcone isomerase* (*CHI*), *anthocyanidin reductase* (*ANR*), *flavanone 3-hydroxylase* (*F3H*), *flavanone 3′-hydroxylase* (*F3’H*) *flavonoid 3′,5′-hydroxylase* (*F3′5′H*), *dihydroflavonol 4-reductase* (*DFR*), *anthocyanidin synthase* (*ANS*), and *leucoanthocyanidin reductase* (*LAR*) involved in catechin synthesis. In this study, we constructed a spatial expression atlas of key genes involved in catechin biosynthesis using spatial transcriptomic data, and observed that multiple structural genes exhibited high expression levels in bud tissues (Figs 3b and S6). To further validate this observation, we incorporated tissue-specific transcriptomic data from the ‘Tieguanyin’ cultivar for in-depth analysis (Table S5). The results revealed that core genes in the catechin biosynthetic pathway, such as *CsANR*, *CsLAR*, and *CsDFR*, consistently showed elevated expression in bud tissues. For instance, *CsANR* (*CsTGY08G0000373*) reached an average expression level of 1710.85 in buds, which was substantially higher than in roots (491.6) and flowers (30.4). Similarly, several members of the *CsLAR* gene family exhibited high expression in both buds and young leaves. These findings are consistent with those reported by Zhao *et al*. [16], who demonstrated that flavonoid biosynthetic enzymes are predominantly expressed in the palisade mesophyll cells of tea buds and leaves. In this study, spatially specific expression patterns of these genes were analyzed. The results showed that in Cluster 3 (vascular bundle and spongy mesophyll in leaves), key structural genes such as *DFR*, *F3H*, *F3′5′H*, and *SCPL* were highly expressed, suggesting that the vascular bundle and surrounding spongy mesophyll in leaves may be the primary functional areas for catechin biosynthesis. Additionally, Cluster 5 (xylem, phloem, and vascular bundle in buds) and Cluster 4 (xylem, phloem, spongy mesophyll, and parenchyma in leaves) also exhibited some activity in the expression of structural genes, suggesting their potential involvement in the synthesis of precursor substances or initial metabolic responses, especially upstream genes such as *CHS*, *PAL*, and *4CL*. Clusters 0/6/8 primarily corresponding to early tissues in buds or stems (such as phloem, spongy mesophyll, and xylem), showed low expression levels for the vast majority of structural genes, indicating that these tissues have not significantly activated the catechin biosynthesis pathway or mainly function in material transport. Cluster 1 (epidermal cells, cuticle, collenchyma, and parenchyma in stems) and Cluster 7 (pith) overall exhibited low expression levels, suggesting limited activity of catechin synthesis in these structures, further supporting their spatially specific expression patterns. It is noteworthy that different members of the *SCPL* family showed some expression in both Clusters 1 and 3 but may have functional differentiation, involving tissue-specific roles in catechin modification or transport processes. The above results indicate that the structural genes for catechin synthesis exhibit significant spatial heterogeneity in different tissues of the tea shoot, being highly enriched in Cluster 3 (vascular bundle and spongy mesophyll in leaves), while showing low expression in early differentiated tissues of buds and stems, reflecting that their metabolic activity is closely related to tissue type and demonstrating distinct spatial regulation characteristics.

The expression patterns of structural genes are typically regulated by TFs. Since TFs need to spatially bind to the promoter regions of structural genes to exert regulatory functions, we propose that TFs with spatial expression patterns similar to those of structural genes involved in secondary metabolism may participate in the regulation of secondary metabolites in tea plants. We refer to this as the ‘spatial determinism of regulation’ principle. To further explore these key TFs, according to the expression of genes in different cluster, the genes were grouped by K-means. After filtering the genes with low expression, 17 192 genes were divided into nine groups (Fig. S7 and Table S8). At the same time, the PlantTFDB website (https://planttfdb.gao-lab.org/prediction.php) was used to predict the TFs of ‘Tieguanyin’ genome, and a total of 1028 TFs in nine groups were obtained. Furthermore, the binding sites of the structural genes were predicted, and the TF families and binding sites (Table S9) that might bind to them were obtained. Integrated spatial expression consistency and binding site information, the network of important TFs that may regulate the metabolism of catechins, theanine, and caffeine was drawn. For catechin metabolism, *bHLH* (93 members), *MYB* (64 members), *ERF* (65 members), *WRKY* (39 members), *C2H2* (52 members), and *GRAS* (40 members) demonstrate the highest connectivity, indicating that they may form core transcriptional regulatory modules modulating catechin biosynthesis. These families are widely involved in plant secondary metabolism, hormone response, and stress adaptation, and have been reported to play key roles in the flavonoid and phenylpropanoid pathways [26] (Fig. 3c).

### Spatial expression of genes related to the metabolism of theanine in tea shoot

**Figure 4:**
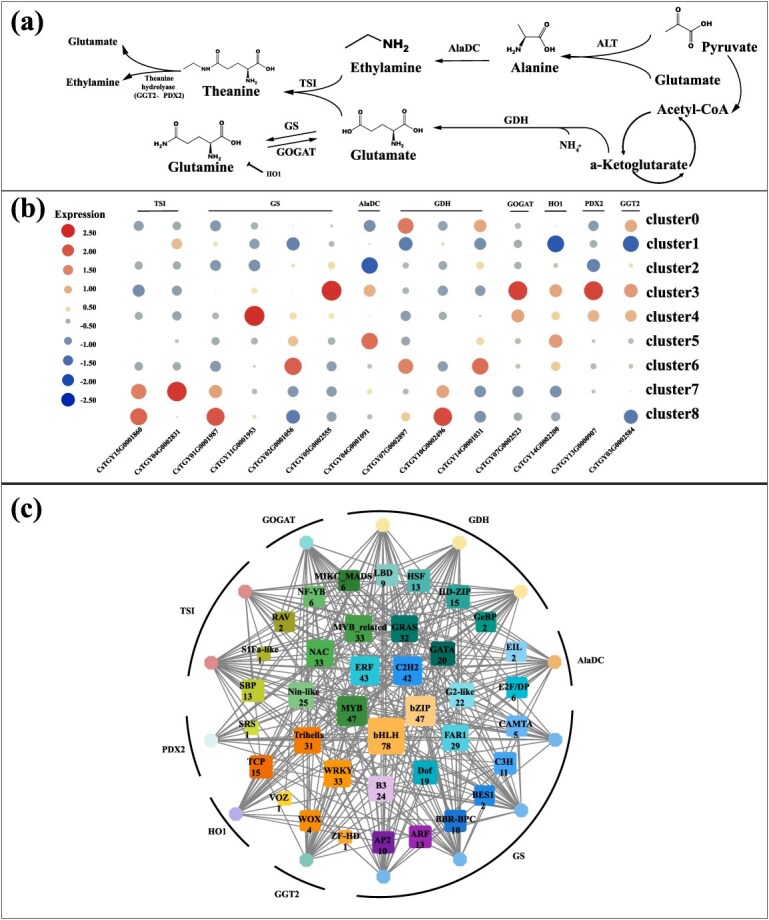
Spatial expression information of relevant structural genes in tea theanine biosynthesis and potential transcriptional regulation networks in tea tender shoot. (a) The biosynthesis pathway of theanine in tea plants. *AlaDC*, *ALT*, *GDH*, *GGT2*, *GOGAT*, *GS*, *HO1*, *PDX2*, and *TSI* represent genes encoding, *alanine decarboxylase*, *alanine aminotransferase*, *glutamate dehydrogenase*, γ-*glutamy*-*transpeptidase*, *glutamate synthase*, *Haem Oxygenase 1*, *pyridoxine biosynthesis 2*, and *theanine synthase*, respectively. (b) Heatmap shows the spatial expression pattern of theanine biosynthesis pathway genes in tea plants. (c) Interaction network between structural genes and potential TFs in the theanine biosynthesis pathway. Based on the coexpression patterns of genes across different groups, TFs potentially involved in regulation were screened by identifying those that exhibited expression trends consistent with the structural genes. The colored octagons represent the structural genes associated with theanine biosynthesis. The color-coded squares represent TFs potentially regulating structural genes, with gray lines depicting regulatory connections between these TFs and structural genes. The numerical annotations indicate the gene count of the corresponding TFs.

The biosynthesis of theanine is primarily mediated by five key enzymes, including theanine synthase (TSI), alanine decarboxylase (AlaDC), glutamine synthase (GS), glutamate dehydrogenase (GDH), and glutamate synthase (GOGAT) (Figs 4a and S8) [27, 28]. The expression of *TSI* genes was mainly confined to Clusters 7 and 8, corresponding to the pith and vascular tissue regions of the stem, with generally weak expression signals across the overall spatial scope. This suggests that the stem may possess certain synthetic potential but is more likely to play a supplementary or localized regulatory role rather than serving as the primary site for theanine synthesis. The biosynthesis of theanine depends on glutamate and ethylamine. The potential synthetic enzyme for ethylamine, AlaDC, shows slightly higher expression mainly in Clusters 3 and 5, corresponding to the vascular tissue of the leaf and the xylem region of the bud, respectively. Among the glutamate-related genes, *GS*, *GOGAT*, and *GDH* exhibit moderately higher expression mainly in Clusters 3, 4, and 6, corresponding primarily to vascular or spongy tissue regions in the leaf and bud, though the overall expression intensity is not high. Together, these results indicate that the enzymatic basis for theanine synthesis substrates is generally expressed at low activity in aerial tissues, consistent with their nonprimary synthetic function. Compared to the synthesis pathway, theanine places more emphasis on being involved in hydrolysis in aerial tissues. The structural gene related to *hydrolysis, γ-glutamy-transpeptidase 2* (*GGT2*), is highly expressed in Clusters 0, 3, and 4, corresponding to the phloem, spongy tissue, and vascular regions of the bud and leaf, which are often involved in material transport and energy allocation. The function of GGT2 is to catalyze the hydrolysis of theanine, releasing glutamate and ethylamine, thereby providing fundamental substrates for nitrogen recycling [29]. The cofactor pyridoxal phosphate required for this reaction is encoded and synthesized by pyridoxine biosynthesis 2 (PDX2) [30], which is also highly expressed in the same Clusters 3 and 4. In addition, *HO1* (*Heme oxygenase 1*) gene also showed an obvious high expression trend in Clusters 3 and 5, corresponding to vascular and xylem regions of leaves and buds, respectively. HO1 has been proven to be a negative regulator of theanine accumulation, which indirectly changes the degradation dynamics of theanine through its influence on glutamate metabolism [31]. In this study, *HO1* partially overlaps with *GGT2* and *PDX2* in space, which further supports its potential role in the steady-state regulation of theanine in leaves. Multitissue transcriptome data from the ‘Tieguanyin’ cultivar also revealed that *GGT2* and *HO1* exhibit higher expression levels in buds and leaves, consistent with their spatial enrichment in aerial tissues observed in this study, thereby supporting the reliability of the expression patterns detected (Table S5).

Coexpression network analysis identified multiple TF families associated with theanine metabolism genes (Fig. 4c). Among them, *bHLH* (78 members), *MYB* (47 members), *ERF* (43 members), *bZIP* (47 members), and *WRKY* (33 members) are the core TF families with the highest connectivity. They extensively regulate key structural genes such as *TSI*, *ADC*, *GS*, and *GGT2*, forming a potential master regulatory network. Notably, the *WRKY* and *MYB* families are particularly densely connected to *ADC* and *AlaDC*, suggesting they may play important roles in amino acid substrate synthesis or metabolic flux regulation. TFs such as *AP2*, *ARF*, *TCP*, *trihelix*, and *FAR1* also appear in the network, implying that theanine synthesis may involve cross-regulatory mechanisms with hormone signaling (e.g., ethylene, ABA, auxin).

### Spatial expression of genes related to the metabolism of caffeine in tea shoot

**Figure 5:**
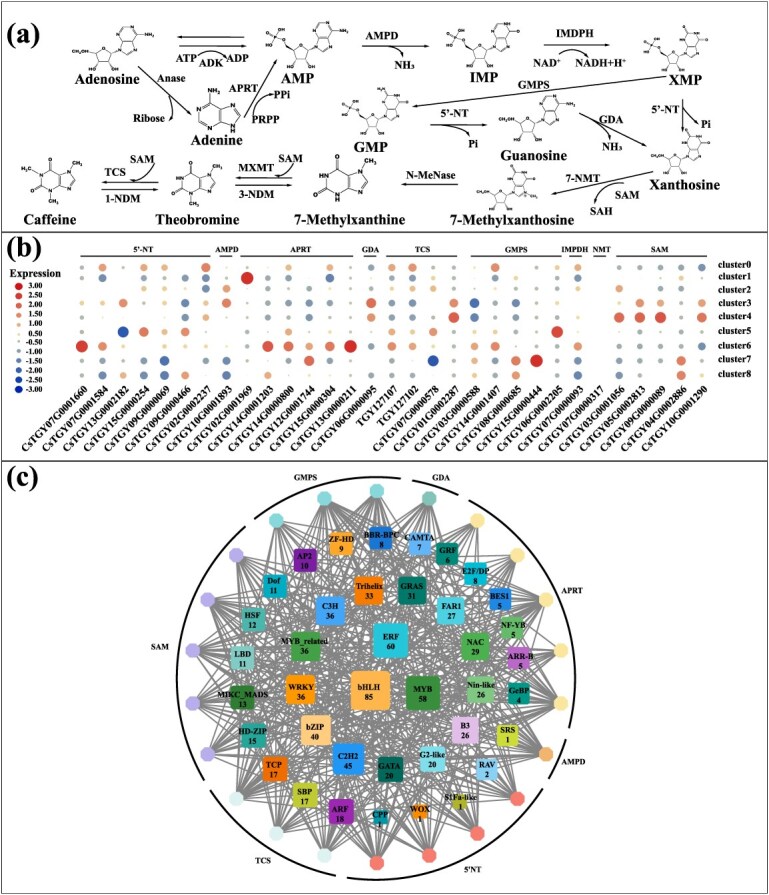
Spatial expression information of relevant structural genes in tea caffeine biosynthesis and potential transcriptional regulation networks in tea tender shoot. (a) The biosynthesis pathway of caffeine in tea plants. 1-*NDM*, 3-*NDM*, *5′-NT*, *7-NMT*, *ADK*, *AMP*, *AMPD*, *Anase*, *APRT*, *GDA*, *GMPS*, *IMP*, *IMPDH*, *MXMT*, N-*MeNase*, *SAH*, *SAM*, *TCS*, and *XMP* represent genes encoding *1 N-demethylase*, *3 N*-*demethylase*, *5′-nucleotidase*, *7-methylxanthosine synthase*, *adenosine kinase*, *adenosine monophosphate*, *AMP deaminase*, *adenosine nucleosidase*, *adenine phosphoribosyltransferase*, *guanine deaminase*, *guanylic acid synthase*, *inosine monophosphate*, *IMP dehydrogenase*, *theobromine synthase*, *N-methylnucleotidase*, *s-adenosyl-L-homocysteine*, *s-adenosyl-L-methionine*, *caffeine synthase*, and *xanthosine monophosphate*, respectively. (b) Heatmap shows the spatial expression pattern of caffeine biosynthesis pathway genes in tea plants. (c) Interaction network between structural genes and potential TFs in the caffeine biosynthesis pathway. Based on the coexpression patterns of genes across different groups, TFs potentially involved in regulation were screened by identifying those that exhibited expression trends consistent with the structural genes. The colored octagons represent the structural genes associated with caffeine biosynthesis. The color-coded squares represent TFs potentially regulating structural genes, with gray lines depicting regulatory connections between these TFs and structural genes. The numerical annotations indicate the gene count of the corresponding TFs.

The biosynthetic pathway of caffeine is characterized by several essential methylation events. These reactions are predominantly mediated by the catalytic functions of xanthine N-methyltransferases and 7-methylxanthine nucleosidase (Figs 5a and S9). The *caffeine synthase* (*TCS*) was predominantly enriched in Clusters 0, 3, 4, 5, and 6 (Fig. 5b). These regions correspond to tissues such as the xylem and phloem in buds, as well as vascular bundles and spongy mesophyll in leaves, indicating that *TCS* collaboratively participates in caffeine biosynthesis across multiple functional zones. In addition to *TCS*, other key structural genes exhibited complementary expression profiles in distinct tissues, revealing a finely partitioned tissue-specific organization of the caffeine synthesis pathway. The *s-adenosyl-L-methionine* (*SAM*) were highly expressed in Clusters 3 and 4, indicating that the vascular bundles and adjacent tissues in leaves serve as the primary sites for generating the methyl donor, thereby directly supporting methylation reactions catalyzed by TCS. Similarly, *guanine deaminase* (*GDA*) showed relatively high expression in Clusters 3 and 4, where it facilitates the deamination of purine substrates, establishing a foundation for subsequent metabolic steps. *Adenine phosphoribosyltransferase* (*APRT*) was primarily expressed in Cluster 6, which corresponds to the xylem and spongy tissue in buds, potentially associated with the recycling of caffeine precursors. GMP demonstrated significant expression in Clusters 6 and 7 (pith region of the stem), suggesting its role in supplying guanylate precursors required for caffeine metabolism within bud and pith tissues. Multi-tissue transcriptome data from Tieguanyin revealed that *TCS* and *SAM* exhibit the highest expression levels in buds, young leaves, and stems, further supporting their coordinated spatial involvement in caffeine metabolism across aerial tissues and reinforcing the reliability of the spatial transcriptomic findings presented in this study (Table S5).

Coexpression network analysis identified multiple TF families significantly correlated with key caffeine metabolism genes (Fig. 5c). Among them, bHLH (85 members), MYB (58 members), ERF (60 members), WRKY (36 members), bZIP (40 members), and C2H2 (45 members) serve as core regulatory hubs in the network, demonstrating broad involvement in regulating key structural genes such as *TCS*, *GDA*, *guanine deaminase*, *guanylic acid synthase* (*GMPS*), *APRT*, and *SAM*. TF families containing domains such as NAC, GRAS, HD-ZIP, Dof, GATA, and FAR1 participate in regulating multiple synthesis/modification genes and may possess tissue-specific or signal-integrating functions in caffeine regulation. TFs including Trihelix, TCP, AP2, ARF, and ZF-HD connect multiple metabolic pathways, suggesting their potential involvement in coordinating developmental processes and caffeine metabolism.

### Identification of CsTCP4, a TF that coregulates both catechins and theanine metabolism

**Figure 6:**
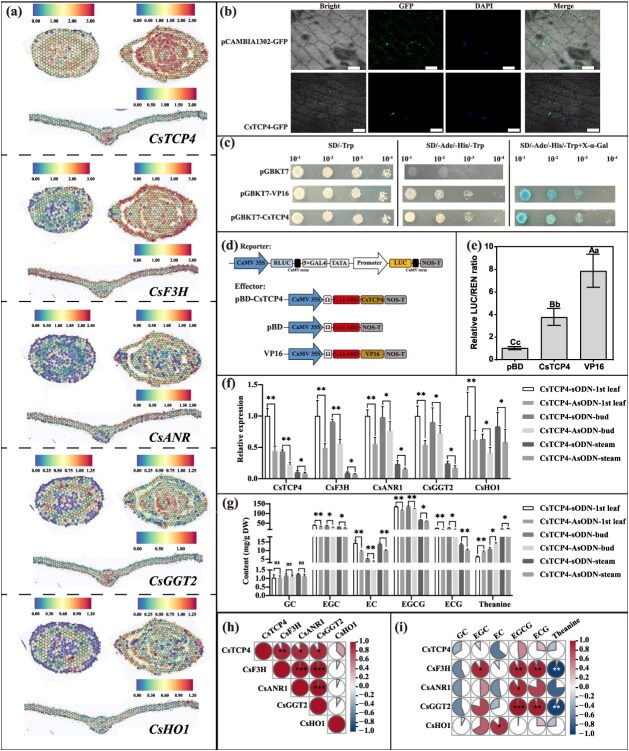
CsTCP4 is a transcriptional activator that can be involved in regulating the metabolism of catechins and theanine in tea plant. (b) Subcellular localization of CsTCP4 in onion epidermis. Scale bar = 100 μm. (c) Transcriptional activity assay of CsTCP4 in yeast Y2HGold strains. (d) Schematic diagrams of the reporter and effecter vectors used in transcriptional activity assay for CsTCP4. (e) Determination of the LUC/REN activity in tobacco leaves. Lowercase and uppercase letters represent *P* < 0.05 and *P* < 0.01, respectively. (f) The expression of *CsTCP4*, *CsF3H*, *CsANR*, *CsGGT2*, and *CsHO1* after the tea shoots were incubated by asODN-CsTCP4. The sODN was the control group. (g) The content of GC, EGC, EC, EGCG, ECG, and theanine after the tea shoots were incubated by asODN-CsTCP4. The sODN was the control group. The ‘*’ and ‘**’ represent *P* < 0.05 and *P* < 0.01, respectively. (h) The Pearson correlation analysis of *CsTCP4*, *CsF3H*, *CsANR*, *CsGGT2*, and *CsHO1* expression in tea bud, leaf, and steam. The ‘*’ and ‘**’ represent *P* < 0.05 and *P* < 0.01, respectively. (i) The Pearson correlation analysis of gene expression and metabolite content in tea bud, leaf, and steam. The ‘*’ and ‘**’ represent *P* < 0.05 and *P* < 0.01, respectively.

Based on the principle of ‘spatial determinism of regulation’, we found that a TCP member (CsTGY07G0000588) exhibited a shared spatial expression pattern with genes involved in both catechins’ synthesis [*CsANR* (CsTGY08G0001693) and *CsF3H* (CsTGY09G0002779)] and theanine hydrolysis [*CsHO1* (CsTGY14G0002200) and *CsGGT2* (CsTGY03G0002584)] (Fig. 6a). Additionally, the promoter regions of these four structural genes contain TCP-binding sites, suggesting that CsTGY07G0000588 may play a role in the coordinated regulation of catechins and theanine metabolism (Table S9). We therefore decided to focus on functional validation of this gene, while also evaluating the effectiveness of ST in elucidating the regulatory mechanisms underlying the synthesis of flavor compounds in tea shoots. The TCP family of *C. sinensis* have been systematically identified [32], according to the BLAST result, CsTGY07G0000588 was identified as *CsTCP4* [10]. To verify whether CsTCP4 has the typical characteristics of TF, we first validated its subcellular localization. The result showed that CsTCP4 localized in the nucleus when comparing the GFP signal of the CsTCP4-GFP with nuclear 4′,6-diamidino-2-phenylindole (DAPI) staining, whereas the GFP signal accumulated throughout whole onion epidermal cells infiltrated with a pCAMBIA1302-GFP vector (Fig. 6b). This finding indicated that CsTCP4 is a nuclear localization protein. To assess the transcriptional activation potential of CsTCP4, the yeast two-hybrid (Y2H) experiment was conducted. We constructed a fusion protein by linking the CDS of *CsTCP4* to the GAL4 DNA-binding domain (GAL4BD). This recombinant plasmid, pGAL4BD-CsTCP4, was subsequently transformed into Y2HGold yeast cells. For comparative analysis, pGAL4BD and pGAL4BD-VP16 were employed as negative and positive controls, respectively. As depicted in Fig. 6c, all transformed yeast strains (pGAL4BD-CsTCP4, pGAL4BD, and pGAL4BD-VP16) exhibited robust growth on synthetic defined medium lacking tryptophan (SD-Trp). However, only the yeast harboring pGAL4BD-CsTCP4 and pGAL4BD-VP16 demonstrated growth on selective medium lacking tryptophan, histidine, and adenine (SD-Trp/His/Ade). Furthermore, these two strains displayed a blue coloration upon exposure to X-α-D-galactoside, indicating they had β-galactosidase activity. To further investigate the transcriptional activity of CsTCP4 in tobacco leaves, a dual-luciferase reporter assay was employed. The CDS of CsTCP4 was cloned into the pBD vector, resulting in the construct pBD-CsTCP4. The empty pBD vector and the VP16 vector served as the negative and positive controls, respectively (Fig. 6d). Following expression in tobacco, the LUC/REN ratio for both pBD-CsTCP4 and VP16 was significantly elevated compared to the empty pBD vector control (Fig. 6e). These results collectively demonstrate that CsTCP4 possesses transactivation capability.

Thereafter, the antisense oligonucleotide (asODN) interference experiment was employed with tea shoot for transient suppression of *CsTCP4* expression. Compared with the senseODN (sODN) control, the expression of *CsTCP4* was significantly inhibited after 48 h of incubation. Meanwhile, the expression of *CsF3H*, *CsANR*, *CsGGT2,* and *CsHO1* was also significantly downregulated (Fig. 6f). We then detected the content of catechins and theanine in both asODN-incubated and sODN-incubated tea shoots (Fig. 6g). Interestingly, the content of the five detectable catechin components (GC, EGC, EC, EGCG, ECG) was significantly reduced in tea leaves incubated with asODN-CsTCP4, while the theanine content changed in the opposite way. These phenomenons suggested that *CsTCP4* has the typical characteristics of TF and may positively regulate the expression of target genes by binding to their *cis*-acting elements. Subsequently, Pearson correlation analysis was performed based on the expression data of *CsTCP4* and its putative target genes (*CsF3H*, *CsANR*, *CsGGT2*, and *CsHO1*) under asODN and sODN treatments. The results showed that *CsTCP4* expression was significantly and positively correlated with *CsF3H*, *CsANR*, and *CsGGT2* (*P* < 0.01), and also exhibited a moderate positive correlation with *CsHO1*, although this was not statistically significant (Fig. 6h). In addition, correlation analysis between gene expression levels and key metabolite contents revealed that *CsF3H* and *CsANR* were positively correlated with various catechin components, whereas *CsGGT2* showed a significant negative correlation with theanine levels (Fig. 6i). *CsHO1* also showed a negative correlation with theanine, but the correlation was not significant, which might be attributed to its broad expression across multiple tissues and its relatively diverse biological functions (Table S5). Taken together, these results suggest that *CsTCP4* may coordinate the metabolic balance between bitterness and umami by promoting catechin biosynthesis via activation of *CsF3H* and *CsANR*, and by enhancing theanine degradation through the activation of *CsGGT2*.

### CsTCP4 can promote the expression of *CsANR*, *CsF3H*, *CsHO1*, and *CsGGT2* genes by combining with their promoters

**Figure 7:**
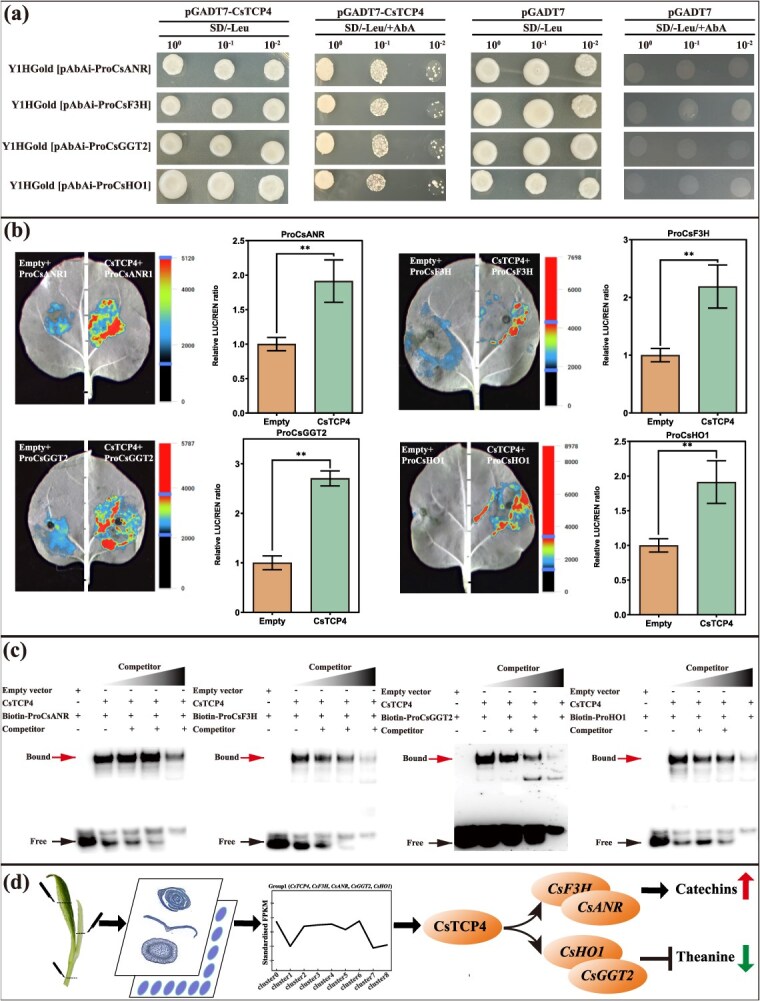
CsTCP4 directly targets and binds to the promoters of *CsF3H*, *CsANR*, *CsHO1*, and *CsGGT2* and regulates their expression. (a) Y1H assay was used to verify that CsTCP4 bonds to the promoters of *CsF3H*, *CsANR*, *CsHO1*, and *CsGGT2*. The pGADT7 empty vector served as a negative control. (b) LUC assays verified the transcriptional activation ability of CsTCP4 toward the *CsF3H*, *CsANR*, *CsHO1*, and *CsGGT2*. Luminescence imager was used to capture luminous images. The ‘**’ represent *P* < 0.01. (c) Identification of the TCP binding sites recognized by CsTCP4 in the promoter region of *CsF3H*, *CsANR*, *CsHO1*, and *CsGGT2* by EMSA. (d) A model for the mechanism by which CsTCP4 affects catechins and theanine metabolism in tea shoot.

To confirm the potential synergistic regulation of theanine and catechin metabolism by CsTCP4 via direct target to key genes, we performed yeast one-hybrid (Y1H) assays to test its interaction with specific promoter motifs of *CsANR*, *CsF3H*, *CsHO1*, and *CsGGT2*. The yeast strains harboring the pGADT7-CsTCP4 recombinant demonstrated robust growth on both SD/−Leu medium and SD/−Leu supplemented with varying concentrations of aureobasidin A (AbA). In contrast, the control strains containing the empty pGADT7 vector exhibited growth exclusively on SD/−Leu medium, with no growth observed on SD/−Leu containing AbA (Fig. 7a). This suggest that CsTCP4 possesses the ability to bind its targets’ promoter regions. To further investigate the regulatory role of CsTCP4 in the expression of *CsANR*, *CsF3H*, *CsHO1*, and *CsGGT2*, we employed a dual-luciferase reporter assay. Effector and reporter constructs were generated and transiently coexpressed in *Nicotiana tabacum* leaves, as illustrated in Fig. 7b. Comparative analysis revealed that the CaMV35S::CsTCP4 construct significantly enhanced the LUC/REN ratio compared to the control empty vector when cotransfected with reporter plasmids containing the promoter regions of *CsANR*, *CsF3H*, *CsHO1*, and *CsGGT2* genes, which suggested that CsTCP4 acts as a transcriptional activator of these genes. Besides, DNA mobility shift assay (EMSA) was performed to confirm the interaction between CsTCP4 and the binding sites in the *CsANR*, *CsF3H*, *CsHO1*, and *CsGGT2* promoter regions. It showed that CsTCP4 could bind to the four sites (Fig. 7c). Collectively, the data provide evidence that CsTCP4 functions as a transcriptional activator by specifically binding to the promoters of target genes (Fig. 7d).

## Discussion

### Spatial dissection of organ differentiation and transcriptional regulation in tea shoots

The results of this study demonstrated a high consistency between histological zoning and transcriptomic clustering. Clusters 1, 7, and 8 in the stem tissue corresponded to the epidermis/mechanical support tissue, pith, and xylem and phloem, respectively, revealing their functional differentiation in mechanical support, storage, and substance transport. In leaf tissue, Clusters 3 and 4 corresponded to photosynthetic tissues and the vascular system, respectively, clarifying the spatial partitioning of photosynthetic product synthesis and conduction. Bud tissue exhibited higher heterogeneity, with multiple clusters encompassing undifferentiated cells, vascular tissues, and metabolically active cell populations. These results indicate that different organs of the tea plant possess significant functional specificity and complementarity at the spatial level, echoing the ‘nutrient transport-storage partitioning’ pattern previously identified in the grains of cereal crops such as maize and barley [33–35].

Pseudotime trajectory analysis further revealed the dynamic functional state transitions during the development of bud and leaf tissues. A continuous temporal trajectory was formed, transitioning from early unspecialized cell populations to intermediate states involving cell wall remodeling and transmembrane transport, and finally to the establishment of late-stage functions such as photosynthesis, energy metabolism, and defense. Similar differentiation processes were also reflected in ST studies of maize and barley grains, where the endosperm, embryo, and surrounding conductive tissues exhibited distinct temporal gene expression patterns during development, thereby collectively accomplishing substance accumulation and transport [33–35]. This suggests conservation in the spatial regulation of developmental dynamics and functional establishment across different plant organs.

In comparison with the multiorgan single-nucleus transcriptomic atlas established by Guo *et al*. [36] in *A. thaliana*, our study similarly identified multiple fundamental cell types in tea tender shoots. By integrating spatial clustering with histological annotation, we further delineated functional partitioning among distinct tissues in the tea shoot. With respect to metabolic regulation, catechin biosynthesis-related genes were found to be highly expressed in both the bud and young leaf tissues, exhibiting relatively enriched expression patterns in the palisade mesophyll and perivascular regions. Genes involved in theanine hydrolysis also showed active expression in buds and leaves, displaying clear tissue specificity. This spatially restricted expression pattern is reminiscent of the chlorenchyma cell-mediated shoot regeneration mechanism described by Song *et al*. [19] in tomato callus, suggesting that cell type-specific localization of metabolic regulation may represent a common strategy in plants. Regarding developmental heterogeneity, our pseudotime trajectory analysis revealed the spatial progression of leaf cells from proliferation to differentiation. This finding aligns with the developmental trajectories reconstructed using Stereo-seq in maize inflorescence meristems by Wang *et al*. [34] further supporting the critical role of spatial context in cell fate determination. Collectively, our study extends previous findings by uncovering the spatial regulation of specialized metabolites and developmental programs in tea—a nonmodel species—thereby providing a valuable reference for dissecting tissue development and function in perennial woody crops.

Furthermore, given that the biological regulation of TFs on target genes relies on physical spatial contacts, ST resolving gene expression patterns within specific tissue contexts provides a novel perspective for identifying TF-target interaction modules. In the present study, coexpression network analysis revealed that TF families including bHLH, MYB, ERF, WRKY, bZIP, C2H2, and GRAS acted as highly connected hub nodes across all three metabolic pathways. The crucial roles of these families in flavonoid and phenylpropanoid pathways, as well as in stress response, have been well documented, e.g., the MYB-bHLH-WD40 complex is a core regulatory module for flavonoid synthesis [37]; some MYB and C2H2 zinc finger TFs directly participate in phenylpropanoid metabolism and isoflavone accumulation [38]; WRKY TFs have been confirmed to regulate phenylpropanoid-related genes and enhance plant disease resistance [39]. This suggests that these TFs may constitute key regulatory modules modulating secondary metabolism.

The ‘spatial determinism of regulation’ principle proposed in this study offers a spatially informed strategy for uncovering potential transcriptional regulatory relationships in complex plant tissues. It is grounded in the core assumption of spatial transcriptomics: TFs and their target genes that exhibit strong spatial colocalization and coexpression within tissues are more likely to participate in functional regulatory modules. This principle facilitates the hypothesis-driven prioritization of TF–target pairs by leveraging spatial information to refine candidate selection for functional validation. Nevertheless, spatial colocalization alone does not necessarily imply direct transcriptional regulation. Overlapping spatial expression patterns may also result from shared responses to upstream signals—such as hormonal gradients or environmental stimuli—rather than from direct *cis*-regulatory interactions. Moreover, the resolution limitations of current spatial transcriptomic platforms, where individual capture spots often encompass multiple cells, coupled with inherent biological noise, can introduce false-positive signals in coexpression-based predictions. To mitigate these limitations, we incorporated *cis*-regulatory element analysis to support spatial coexpression findings and retained only TF–target pairs with high spatial correlation within defined tissue clusters. This dual-criteria approach improves both the specificity and reliability of inferred regulatory interactions. Therefore, the ‘spatial determinism of regulation’ should be interpreted not as a tool for causal inference, but as a guiding framework to inform the dissection of tissue-specific regulatory logic. As spatial omics technologies advance, the integration of high-resolution spatial single-cell sequencing, multiomics datasets, and network modeling will further enhance the precision and applicability of this principle in decoding the spatial architecture of plant metabolic regulation.

Although the 10× Visium platform successfully resolved tissue-level gene expression patterns, its inherent spatial resolution imposes limitations. Each capture spot (diameter ~55 μm) contains multiple cells (typically 1–10), resulting in averaged expression signals that may obscure cell type-specific features, particularly for closely adjacent cell types such as sieve elements and companion cells in the phloem, or for distinct palisade cell subtypes in the mesophyll. This ‘spot-averaging’ effect can mask the spatial heterogeneity of rare or low-abundance transcripts, including transcription factors crucial for developmental regulation. Future studies could apply spot deconvolution approaches by integrating single-cell or single-nucleus RNA-seq datasets to infer and computationally separate cellular compositions within individual spots. In addition, next-generation high-resolution spatial techniques (e.g., Stereo-seq, Slide-seqV2) could further resolve transcriptional heterogeneity at the subcellular scale. Combining these with spatially resolved validation techniques, such as smFISH, MERFISH, or immunofluorescence, would provide direct evidence for spatial localization of low-abundance regulatory genes. Ultimately, multiscale integration of spatial omics will enable the transition from tissue-level to single-cell and subcellular-resolution mapping, thereby advancing our understanding of the fine regulatory mechanisms underlying characteristic metabolite biosynthesis in tea.

### CsTCP4 may be a key regulator for balancing the bitterness and umami flavor of tea

The TCP TF family plays important roles in plant organ development, cell division, stress responses, hormone signal transduction, and has increasingly been shown to participate in the regulation of secondary metabolism in recent years [40–42]. In tea plants, studies have reported that CsTCP3/4 can interact with the MBW complex to promote the expression of genes related to catechin synthesis [10]. Based on ST, this study was the first to clarify the dual regulatory role of CsTCP4 in the catechins and theanine metabolic pathways, and a series of functional validation experiments have confirmed its characteristics as a ‘metabolic crossroads regulator’. Traditionally, mining key genes involved in the synthesis and regulation of flavor metabolites has relied heavily on the accumulation and differential analysis of large amounts of RNA-seq data [25]. However, this method provides transcriptomic profiles at the tissue-averaged level, making it difficult to reveal gene-specific expression features in particular cell types or tissue compartments, leading to the potential masking of some low-abundance or spatially specific genes. The ST sequencing used in this study, combined with the ‘spatial regulation principle’, allowed for the rapid and precise identification of target transcription factors within the context of tissue spatial background. For example, our ST analysis revealed that the spatial expression pattern of *CsTCP4* significantly overlaps with that of catechin synthesis genes (*CsANR*, *CsF3H*) and theanine degradation genes (*CsHO1*, *CsGGT2*), directly suggesting its potential regulatory role. This advantage fully demonstrates the unique value of ST technology in functional gene discovery.

At the spatial level, the *CsTCP4* exhibits high expression in both bud and leaf tissues, suggesting that it plays an important role in these metabolically active organs. Functional validation experiments showed that CsTCP4 can directly bind to and activate the promoters of the aforementioned structural genes, thereby achieving coordinated regulation of catechin accumulation and theanine hydrolysis (Figs 6 and 7). When *CsTCP4* expression was inhibited via asODN, the content of five major catechins (GC, EGC, EC, EGCG, ECG) in the tea leaves significantly decreased, while the theanine level markedly increased. This result indicates that *CsTCP4* plays a key role in the balance between ‘bitterness’ and ‘umami’ flavors; i.e., by promoting catechin synthesis and accelerating theanine degradation, it shifts the tea flavor profile toward a ‘bitter and astringent’ direction. This not only provides new experimental evidence for the spatial regulation mechanism of flavor metabolism in tea plants but also offers a potential molecular target for improving tea quality.

From a broader perspective, the discovery of CsTCP4 as a metabolic crossroads regulator highlights an important frontier for future research—deciphering the upstream signals that govern its expression and activity [43]. Although our spatial transcriptomic analysis establishes CsTCP4 as a key coordinator of catechin biosynthesis and theanine degradation, the regulatory hierarchy controlling this transcription factor remains largely unknown. Future studies should therefore focus on dissecting whether CsTCP4 is activated by developmental programs, hormone-mediated pathways (such as auxin, jasmonate, cytokinin, or ABA signaling), or environmental cues including light quality, nitrogen availability, or stress stimuli. To address these questions, several complementary approaches will be particularly valuable. First, comprehensive *cis*-element profiling of the CsTCP4 promoter combined with DAP-seq or CUT&Tag assays can help identify upstream transcription factors that directly bind and regulate CsTCP4. Second, integrating large-scale transcriptome datasets, hormone treatment experiments, and coexpression network analyses could reveal whether CsTCP4 acts downstream of specific hormonal signaling modules. Third, spatial correlation analysis within the ST dataset may uncover whether *CsTCP4* expression gradients align with developmental axes or tissue-specific metabolic domains. Together, these strategies will enable the construction of a multilayered regulatory framework that explains how CsTCP4 is positioned within the broader signaling landscape and how its activity is fine-tuned to shape the balance between bitterness and umami in tea shoots. In summary, the functional validation of CsTCP4 not only uncovers a new regulatory layer in the flavor metabolic network of tea plants but also demonstrates the great potential of spatial transcriptomic technology in functional gene mining. It provides a theoretical basis and practical direction for achieving precise balance between bitterness and umami through molecular breeding.

## Materials and methods

### Plant materials and tissue embedding

The tender shoots (including tender stems, leaves, and buds) of *C*. *sinensis* ‘Tieguanyin’ trees planted in the greenhouse of Fujian Agriculture and Forestry University, Fuzhou, China (26°05*′*N, 119°18*′*E) were used as experimental materials for ST analysis. To obtain high-quality tea tissue sections, we optimized the embedding process related to the description of Giacomello *et al*. [44]. For the embedding of tea apical buds and tender leaves, the tissues were immersed in 1 ml 0.05% tween 20 for 15 min under room temperature, then were transferred into a centrifuge tube with precooled 75% OCT, vacuumed for 10 min at room temperature. The OCT on the surface of the tissues was gently dried and then they were placed in precooled OCT-containing embedding boxes, and the position of the tissues was adjusted to ensure proper sectioning planes. Thereafter, the bottoms of the embedding boxes were immersed in isopentane precooled with liquid nitrogen. When the OCT wrapped around the tissues was completely frozen, the embedding boxes were stored at −80°C until further analyses.

For the embedding of tea tender stems, the tissues were immersed in 1 ml 0.05% tween 20 for 15 min under room temperature, then were immersed in 1 ml of precooled EAA (absolute ethanol:acetic acid = 3:1) and vacuumed for 10 min to fix the tissues. The fixed tissues were transferred into 1 ml of precooled 10% sucrose solution, vacuumed for 20 min, and then the sample is shaken at 4°C until the tissues settled to the centrifuge tube bottom. The 10% sucrose solution was replaced by 20% sucrose solution, and the fixed tissues were vacuumed and shaken again as before. Thereafter, the 20% sucrose solution on the surface of the tissues was gently dried and then they were transferred into a precooled OCT-containing embedding box, and the position of the tissues was adjusted to ensure proper sectioning planes. The bottoms of the embedding boxes were immersed in isopentane precooled with liquid nitrogen. When the OCT wrapped around the tissues was completely frozen, the embedding boxes were stored at −80°C until required. All the cryoblocks were sliced at −20°C cryo-chamber, and a section thickness of 15 μm was adopted for all tissues.

### Tissue optimization

As the permeabilization time varies from one tissue to another, tissue optimization is required to determine the optimal permeabilization time in the construction of spatial gene expression library before starting formal experiments [35]. Firstly, the cryo-sections were mounted onto the 10× Visium slides (each square area is 6.5 × 6.5 mm^2^, including 5000 barcoded spots with a diameter of 55 μm). The sections were fixed for 30 min with 3.7%–3.8% formaldehyde, and then washed in 1 × phosphate buffer saline for hematoxylin and eosin staining (H&E) for 30 s. Then the slides were cleaned using nuclear-free water and air-drying and were mounted with 85% glycerol and coverslips. Bright-field images were taken at 20× magnification using Metafer Slide Scanning platform (MetaSystems). Secondly, permeabilization with different time gradients and fluorescence-labeled cDNA synthesis were carried out. Finally, the tissues were removed for fluorescence imaging, and the optimal permeabilization conditions were determined by the intensity and dispersion degree of fluorescence signals, i.e., the maximum intensity of the fluorescence signal and no dispersion was the optimal permeabilization time, which was used for the construction of the ST sequencing library.

### Library construction and ST sequencing

Same steps as tissue optimization, tissue sections were affixed onto gene expression slides for fixation, H&E staining, and bright-field imaging. The cDNA was then synthesized from the captured mRNA and prepared into a sequencing library following previously described [45]. Supernatants containing the released cDNA were collected and transferred into 96-well plates, and ST sequencing libraries were prepared using an automated MBS 8000 system [46]. Paired-end sequencing was performed on an Illumina NovaSeq platform (Illumina, CA, USA).

### Analysis of ST data and cell clustering

After quality control, the raw reads were demultiplexed, assigned to their derived spots, and generated a feature-spot matrix according to the barcode oligonucleotides and bright-field microscope images using Space Ranger (v.2.0.0) software from 10× Genomics. The gene expression was counted based on the ‘Tieguanyin’ genome annotation [22]. Sequenced reads were trimmed by Space Ranger of 10× Genomics and mapped to the reference genome using STAR software [47]. The quality of spatial sequencing data is assessed based on the results of Space Ranger analysis and is usually assessed by the median Genes per Spot, Reads Mapped to Geneome, Reads Mapped Confidently to Transcriptome, and the spatial distribution map of tissue sections. To evaluate the stability and reliability of ST data in the absence of multiple biological replicates, three structurally intact and anatomically similar regions were selected from each ST slide corresponding to stem, leaf, and bud tissues for whole-transcriptome correlation analysis.

To get the expression of each spot gene, the reliably compared reads with the same barcode, UMI, and gene annotations were divided into a group. The duplicate UMIs of the genes under each barcode were removed, and the reads of each spot were effectively differentiated by image processing to show the captured region of the organization in the chip and the Spatial Barcode spatial locus information. Then, statistics on the number of Reads in the spots, the median number of genes detected, the UMIs, and the sequencing saturation indicate the gene expression in each spot.

To analyze high-resolution ST data, an unbiased and graph-based clustering of spatial features with the Louvain Method was performed, as previously described [40, 48]. Subsequently, t-SNE [49] clustering analyses were performed using Seurat (v.4.0.1), and the spot clustering results were visually presented in 2D space.

Monocle (v.2.22.0) with the default parameters were used to analyze the spatio-temporal trajectory of tea buds and leaves, and the low-dimensional development trajectory was constructed by DDRTree, and the key differential genes in the development branches were identified based on BEAM model. The initial cell is determined by tissue localization and marker expression. All visualizations show the dynamic evolution process of cell development path and functional transcription module through UMAP and thermogram. The differentially expressed genes in eac cluster were analyzed by TBtools-II (v.2.333) [50], and significant pathways were screened by Fisher test and FDR correction (*P* < 0.05). The K-Means function in R package was used to group the standardized expression data (K = 9), and the scale parameter was adjusted to 0.6, so as to obtain the coexpression pattern of genes in the spatial transcription group, and the subsequent interpretation was combined with functional annotation.

The genes encoding enzymes in the metabolic pathways of catechins, theanine, and caffeine, theanine have been identified [15, 23, 29, 51]. The potential TF-binding sites in the promoter regions of structural genes involved in the biosynthesis of tea characteristic metabolites were predicted by the ‘Binding Site Prediction’ tool of PlantRegMap (https://plantregmap.gao-lab.org/) [52].

### FISH in tissues

To investigate the spatial expression pattern of specific genes during bud development, a marker gene with tissue-specific expression was selected based on spatial transcriptome data. FISH was conducted to localize the expression of this gene in buds at different developmental stages. A FAM-labeled antisense probe targeting the marker gene was used for detection, while a sense probe served as a negative control. All probes were synthesized by Gefan Biotechnology Company (Shanghai, China). Bud samples from various developmental stages were fixed in 4% paraformaldehyde at 4°C overnight. After fixation, the tissues were dehydrated, embedded, and sectioned, followed by prehybridization treatments according to standard FISH protocols. Hybridization was carried out at 37°C for 24 h [53]. Posthybridization, the samples were washed with 1× phosphate-buffered saline (PBS, pH 7.2) containing 5% Triton X-100 and stained with DAPI at room temperature for 30 min to visualize nuclei. Fluorescence signals were captured using a Nikon Eclipse Ci microscope (Japan). The sequences and details of the probes are listed in Table S10.

### RNA and gDNA extraction as well as cDNA synthesis

Total RNA was extracted from tea leaves using the TransZol UP (TransGen, Beijing, China). The quality of the isolated RNA was evaluated through gel electrophoresis and quantified using the Ultramicro spectrophotometer (UNano-1000, UMI Instruments Co., Ltd., Hangzhou, China). RNA samples meeting the quality criteria were subsequently reverse-transcribed into first-strand cDNA using the EasyScript First-Strand cDNA Synthesis SuperMix (TransGen Biotech). The gDNA was extracted from tea shoots of ‘Tieguanyin’ plants using the EasyPure Genomic DNA Kit (TransGen Biotech).

### Suppression of *CsTCP4* in tea leaves

The asODN inhibition was performed to transiently suppress *CsTCP4* expression in tea leaves. The asODN sequence was designed using the Soligo website (https://sfold.wadsworth.org/cgi-bin/soligo.pl), and the control primer was the sODN. The specific sequences were synthesized by GenePharma Co., LTD (Shanghai, China). The tea tender shoots with one bud and one shoot were isolated to soak in 1 ml of 25 μM CsTCP4-asODN and sODN solution, respectively. After soaking for 48 h, the shoots were harvested for detecting related gene expression and the content of catechins and theanine. At least three biological replicates were included. Unless otherwise specified, all primer information used for this study was listed in Table S10.

### qRT-PCR assay

The quantitative real-time PCR (qRT-PCR) was conducted on the DLAB Accurate 96 system (DLAB Scientific Co., Ltd., Beijing, China), following the previously described reaction conditions and mixture [54]. The *CsActin* and *CsGAPDH* genes were served as the internal controls, and relative expression levels of genes were calculated using 2^−ΔΔCT^ method [55].

### Determination of the content of catechins and theanine

The measurement of catechins was performed in accordance with the Chinese national standard GB/T 8313-2018 (Determination of total polyphenols and catechins content in tea), while theanine levels were quantified employing GB/T 23193–2017 (Determination of theanine in tea—using high-performance liquid chromatography). The instrument model of HPLC used in this study is Waters E2695 (Milford, MA, USA).

### Subcellular localization

The CDS of *CsTCP4*, excluding the termination codon, was PCR-amplified and seamlessly cloned into the pCAMBIA1302 vector (containing GFP). The resulting construct 35S::CsTCP4::GFP and control vector pCAMBIA1302-GFP were then introduced into *Agrobacterium tumefaciens* strain EHA105, respectively, and subsequently injected into onion (*Allium cepa* L.) epidermal cells as previously described [56]. Nuclei were visualized by staining with DAPI, and GFP fluorescence was detected using a laser scanning confocal microscope (FV1200, Olympus Corporation, Tokyo, Japan).

### Validation of transcriptional self-activation of CsTCP4 based on Y2H system

The Y2H operation procedure for verifying the transcription self-activation of CsTCP4 was based on the procedure described in a previous study [57]. The CDS of *CsTCP4* was PCR-amplified and integrated into the pGBKT7 vector through seamless cloning, with GAL4 DNA-binding domain (GAL4BD) fused at the N-terminus to form the pGAL4BD-CsTCP4 recombinant. The pGBKT7-VP16 and pGBKT7 vectors were used as positive and negative controls, respectively. Yeast Y2HGold strains were transformed with each vector and incubated on SD/−Trp or SD/−Trp-His-Ade media at 30°C for 2–5 days. If colonies could grow in SD/−Trp-His-Ade media, infiltrate them with X-α-D-galactoside to see if they will turn blue.

### Y1H

The promoter fragments containing the binding motifs of CsTCP4 were PCR-amplified and was inserted into the pAbAi vector between Sma I and Sal I restriction sites. The CDS of *CsTCP4* was inserted into the pGADT7 vector between EcoR I and BamHI restriction sites, generating the prey vector pGADT7-CsTCP4. The Y1H assay was performed following the protocol outlined by previous studies [49, 50, 58, 59] with minor adjustments. The bait constructs were linearized using the BstBI restriction enzyme and individually transformed into Y1HGold yeast strains. These bait-containing yeast strains were cultured on SD/-Ura media, and AbA was applied at varying concentrations (0, 25, 50, 75, 100, 125, and 150 ng·ml^−1^) to identify conditions that inhibited bait yeast growth. The pGADT7-CsTCP4 prey vector was introduced into positive clones using a One Step Brewer’s Yeast Fast Transfer Kit (Protein Interaction Biotech, Xianyang, China) and plated on SD/−Leu or SD/−Leu + AbA media. After incubation at 30°C for ~3 days, the growth of individual colonies was assessed to determine potential interactions between CsTCP4 and the promoter fragments.

### Dual luciferase reporter assay

To evaluate the transcriptional activity of CsTCP4 using the dual luciferase reporter assay, pBD and pVP16 were employed as effector vectors, as described in a previous study [60]. The CDS of *CsTCP4* was cloned into the pBD vector, with the GAL4BD fused at the N-terminus, to generate the pBD-CsTCP4 recombinant construct. The reporter was modified from pGreenII-0800-LUC insert the sequences of 5 × GAL4 and minimal TATA region.

To verify the interaction between CsTCP4 and its targets, the CDS of *CsTCP4* was cloned into the pGreenII 62-SK vector between BamHI and HindIII restriction sites, resulting in the effector construct CaMV35S::CsTCP4. The empty pGreenII 62-SK vector served as the control. The promoter sequences of the target genes were individually cloned into the pGreen-0800-LUC vector to create reporter constructs. Both the effector and reporter vectors were independently introduced into *A. tumefaciens* EHA105 containing pSoup and coinfiltrated into *Nicotiana benthamiana* leaves, following a previously described protocol [61]. After incubation at 25°C for ~48 h, LUC and REN activities were measured using a Dual Luciferase Reporter Gene Assay Kit (Yeasen Biotechnology Co., Ltd., Shanghai, China). Transcriptional activity was determined by calculating the LUC/REN activity ratio. Moreover, the firefly fluorescence was observed using Lumazone digital *in vivo* luminescence imager (Teledyne Imaging, Shanghai, China).

### EMSA

For protein purification, the CDS sequences of *CsTCP4* were cloned into the pMAL-c5X vector containing the maltose-binding protein and expressed in *Escherichia coli* strain Rosetta. Oligonucleotide probes for the recognition of the *CsTCP4* consensus binding sequences derived from four target gene promoters were produced by Tsingke Biotech Co., Ltd. (Fuzhou, China), and these probes were subsequently labeled with 3′ biotin. No labeled probes were used as competitors. The EMSA assay was conducted according to the manufacturer’s instructions from the Chemiluminescent EMSA Kit (Beyotime, Shanghai, China).

## Supplementary Material

Web_Material_uhag003

## Data Availability

The raw sequencing data (spatial transcriptome) reported in this paper have been deposited in the National Genomics Data Center (https://ngdc.cncb.ac.cn/) under project number PRJCA025437.
