# Supplementary material for: Spatial transcriptome analysis of the tea tender shoot sheds light on transcriptional regulation of characteristic metabolites
Source: Hortic Res. 2026 Jan 6;13(4):uhag003. doi: 10.1093/hr/uhag003 (PMC13102515; doi:10.1093/hr/uhag003)
Supplement: Web_Material_uhag003 [file web_material_uhag003.zip › Supplementary Figures.pdf]

Supplementary Figs

Running Title: Spatial transcriptome analysis of the tea tender shoot

Spatial transcriptome analysis of the tea tender shoot sheds light on transcriptional regulation of characteristic metabolites

Cheng Zhang[1,†], Chengzhe Zhou[1,2,†], Caiyun Tian[1], Shengjing Wen[1], Zhendong Zhang[1], Anru Zheng[1], Zhenhan Rui[1], Yuting Li[1], Shuaibo Shao[1], Siwei Deng[1], Zhong Wang[1,2], Yuqiong Guo[1,2,*]

[1]Anxi College of Tea Science, College of Horticulture, Fujian Agriculture and Forestry University, Fuzhou 350002, China

[2]Fujian Collaborative Innovation Center for Green Cultivation and Processing of Tea Tree in Universities, Fujian Agriculture and Forestry University, Anxi County, Quanzhou 362400, China

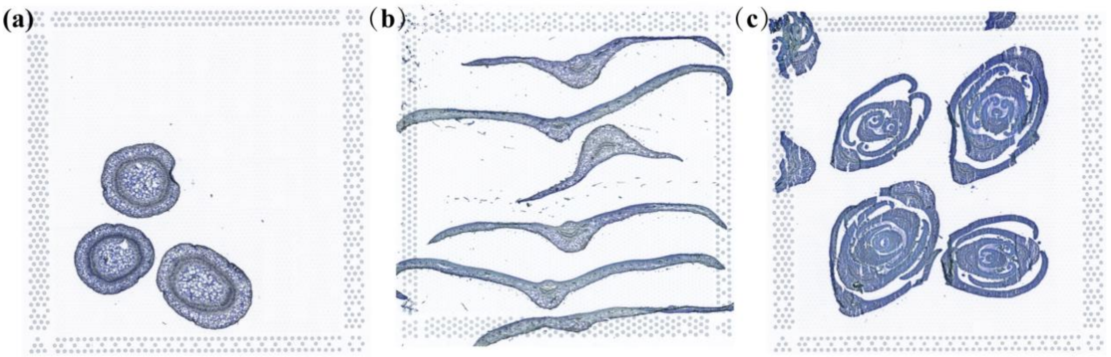

Fig. S1. Schematic illustration of tea plant young shoot tissues mounted on a 10× Visium slide. From left to right: young stem, young leaf, and apical bud. Slide dimensions: 6.5 × 6.5 mm.

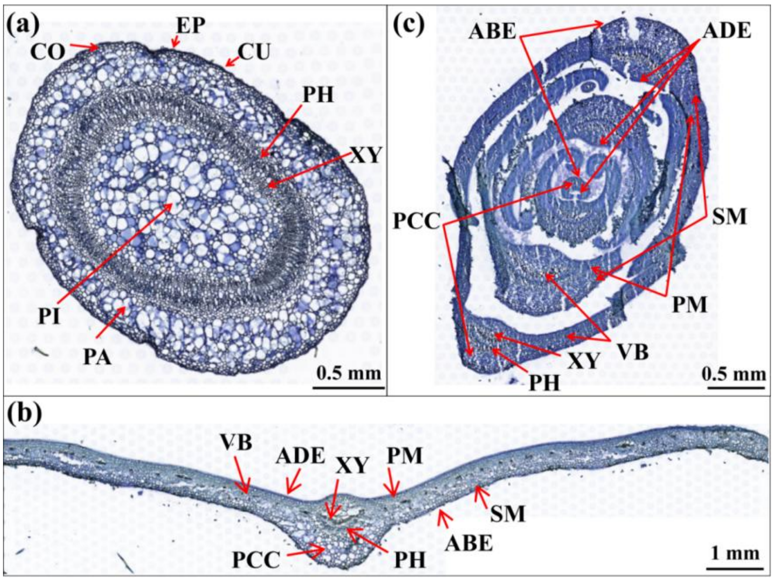

Fig. S2. Transverse section of a young tea shoot for subsequent analysis. (a) Transverse section of stem. (b) Transverse section of leaf. (c) Transverse section of bud. Abbreviation: ABE, abaxial epidermis; ADE, adaxial epidermis; CO, collenchyma; CU, cuticle; EP, epidermis; PA, parenchyma; PCC, parenchyma cells; PH, phloem; PI, Pith; PM, palisade mesophyll; SM, spongy mesophyll;VB, vascular bundle; XY, xylem.

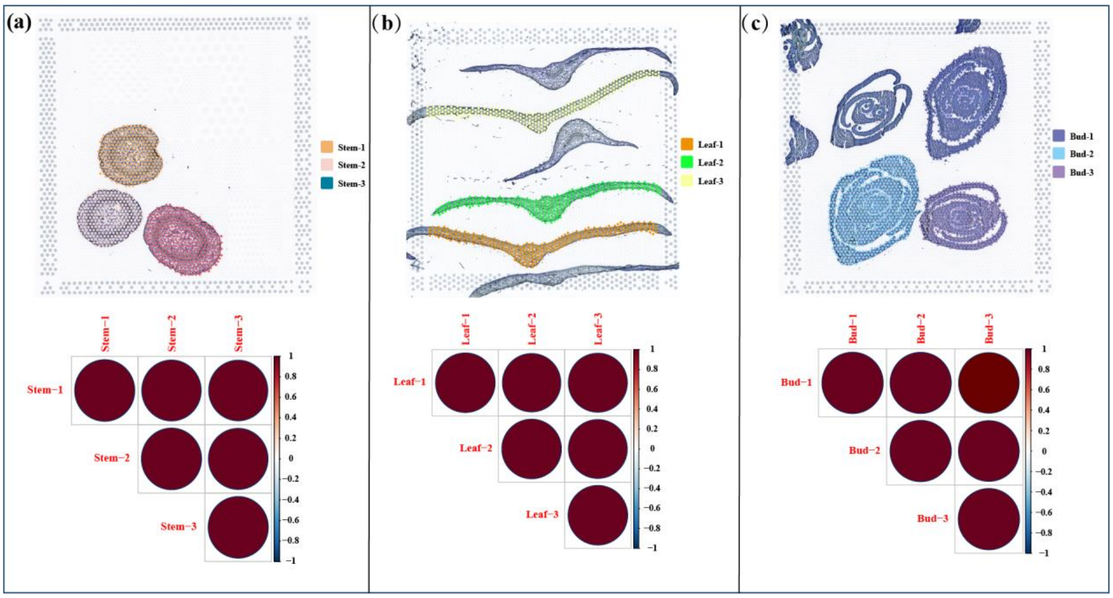

Fig. S3. Transcriptomic correlation analysis among three structurally similar regions within different tender shoot tissues of the tea plant. (a) Stem, (b) leaf, and (c) bud tissues are spatially localized on 10× Visium slides. For each tissue type, three structurally intact and anatomically similar regions (Stem-1 to Stem-3, Leaf-1 to Leaf-3, Bud-1 to Bud-3) were selected to assess data reproducibility. Heatmaps below each panel show the Pearson correlation coefficients (R^2) based on whole-transcriptome expression profiles. All tissue types exhibited extremely high intra-group correlation ($R^2 > 0.98$), indicating excellent technical reproducibility of the ST data at the tissue level.

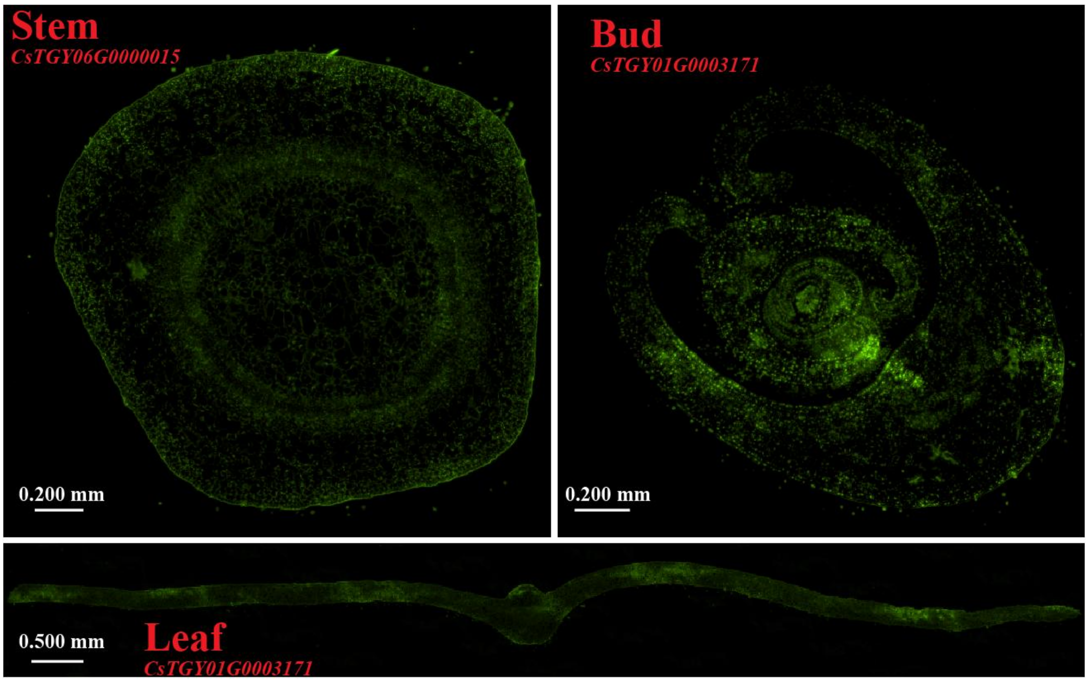

Fig. S4. RNA *in situ* hybridization of candidate marker genes in tea shoot tissues. Scale bars: 0.200 mm (stem, bud), 0.500 mm (leaf).

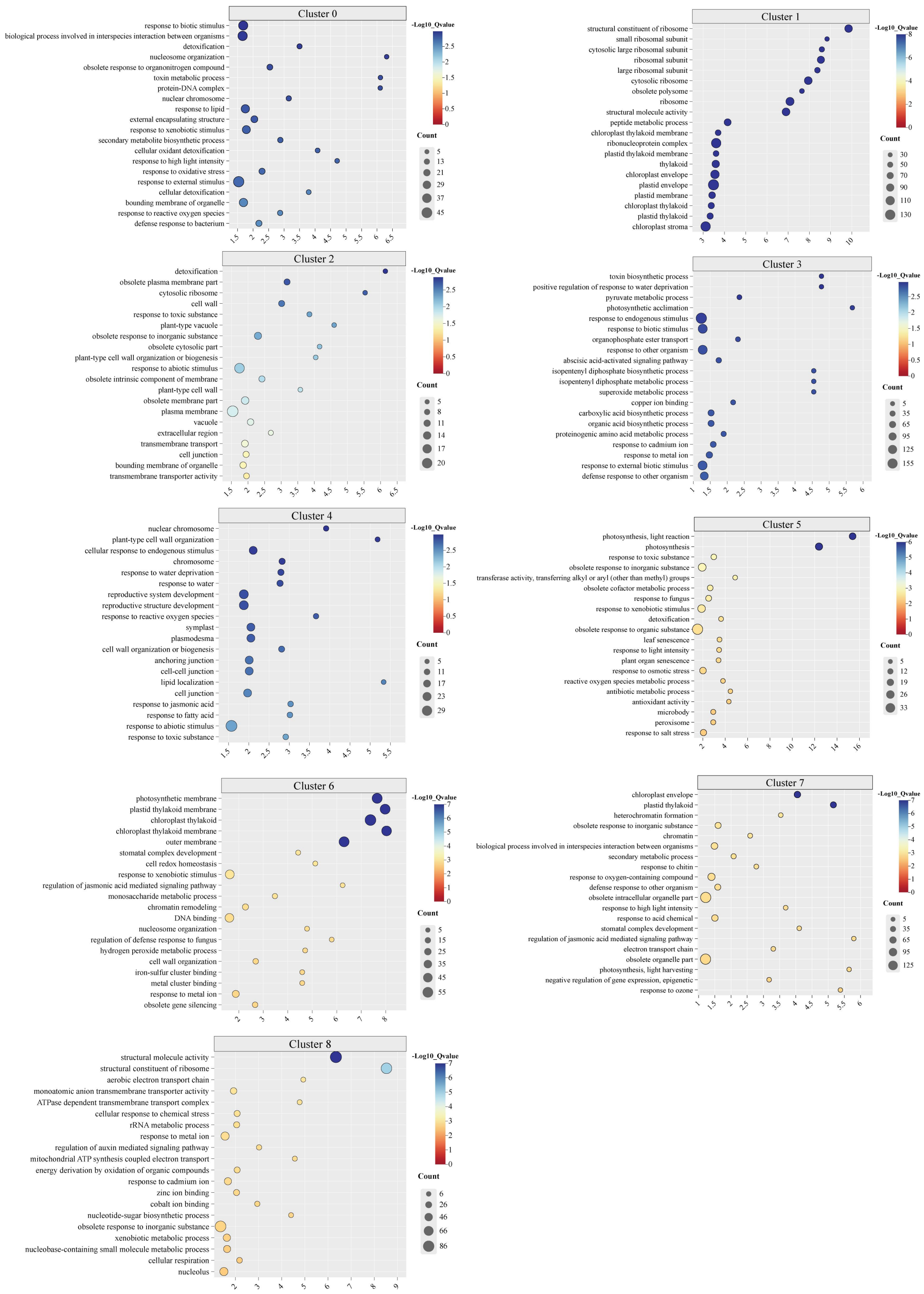

Fig. S5. Gene Ontology (GO) enrichment analysis of marker genes identified across nine cell clusters.

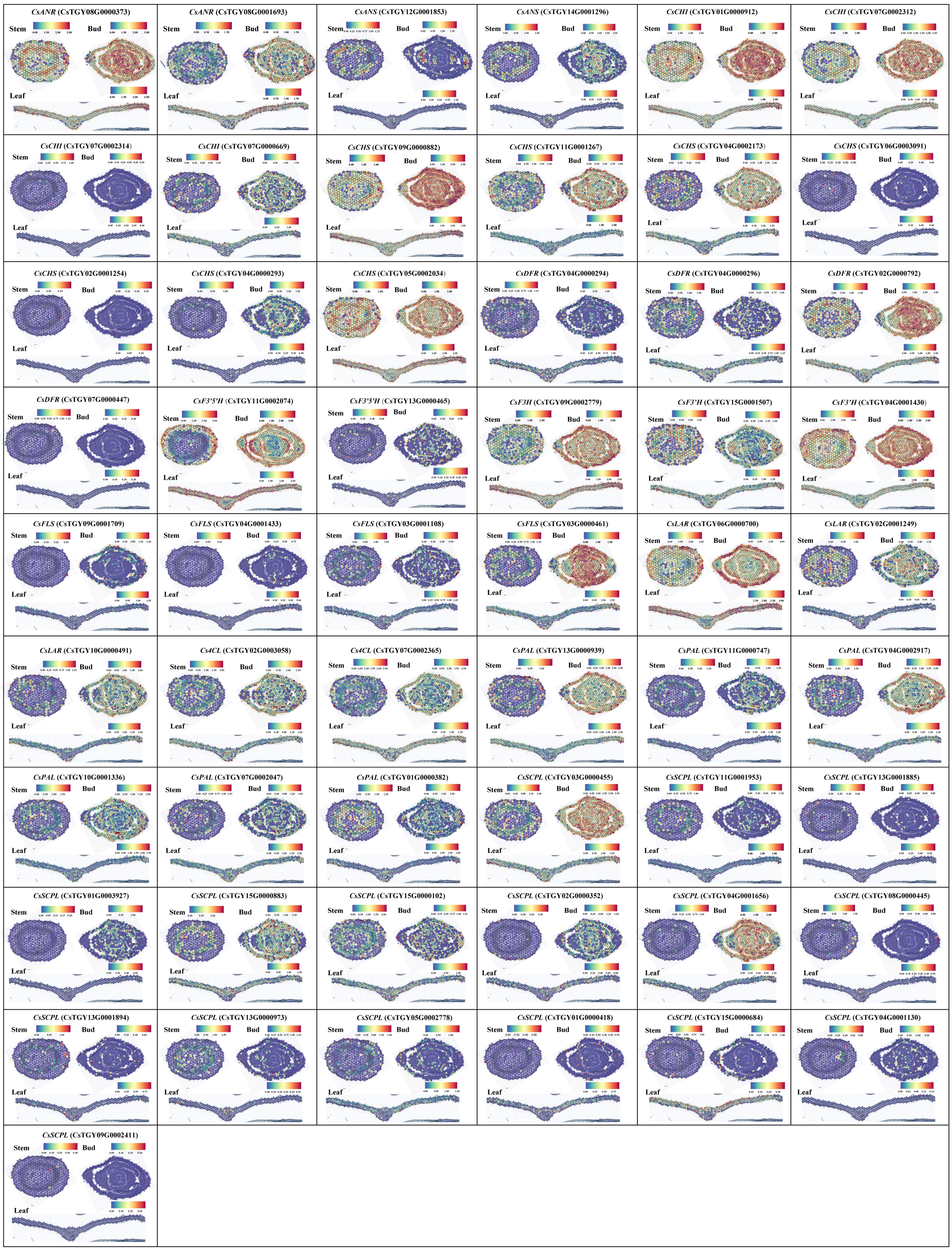

Fig. S6. Spatial expression patterns of structural genes associated with catechin metabolism.

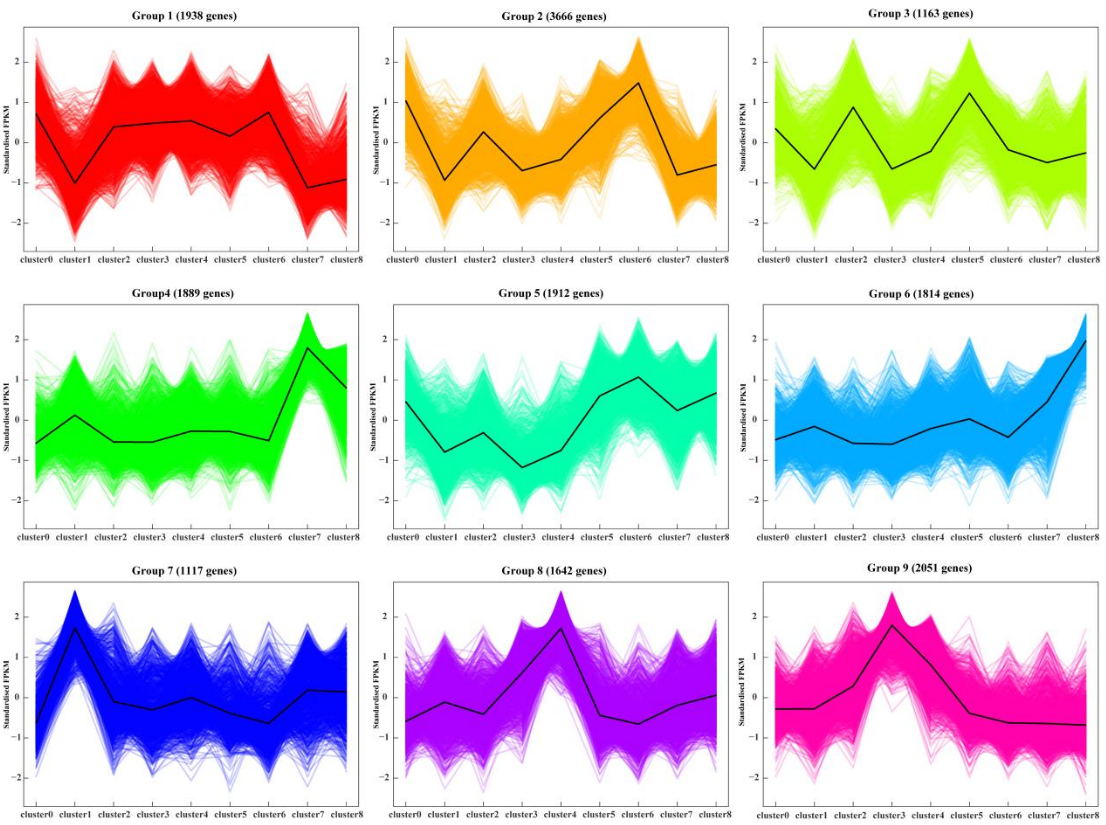

Fig. S7. K-means clustering of gene expression patterns across distinct cell clusters. Categorizing genes into nine groups based on their expression trends in different clusters. The x-axis represents the clusters derived from spot clustering, while the y-axis indicates gene expression values.

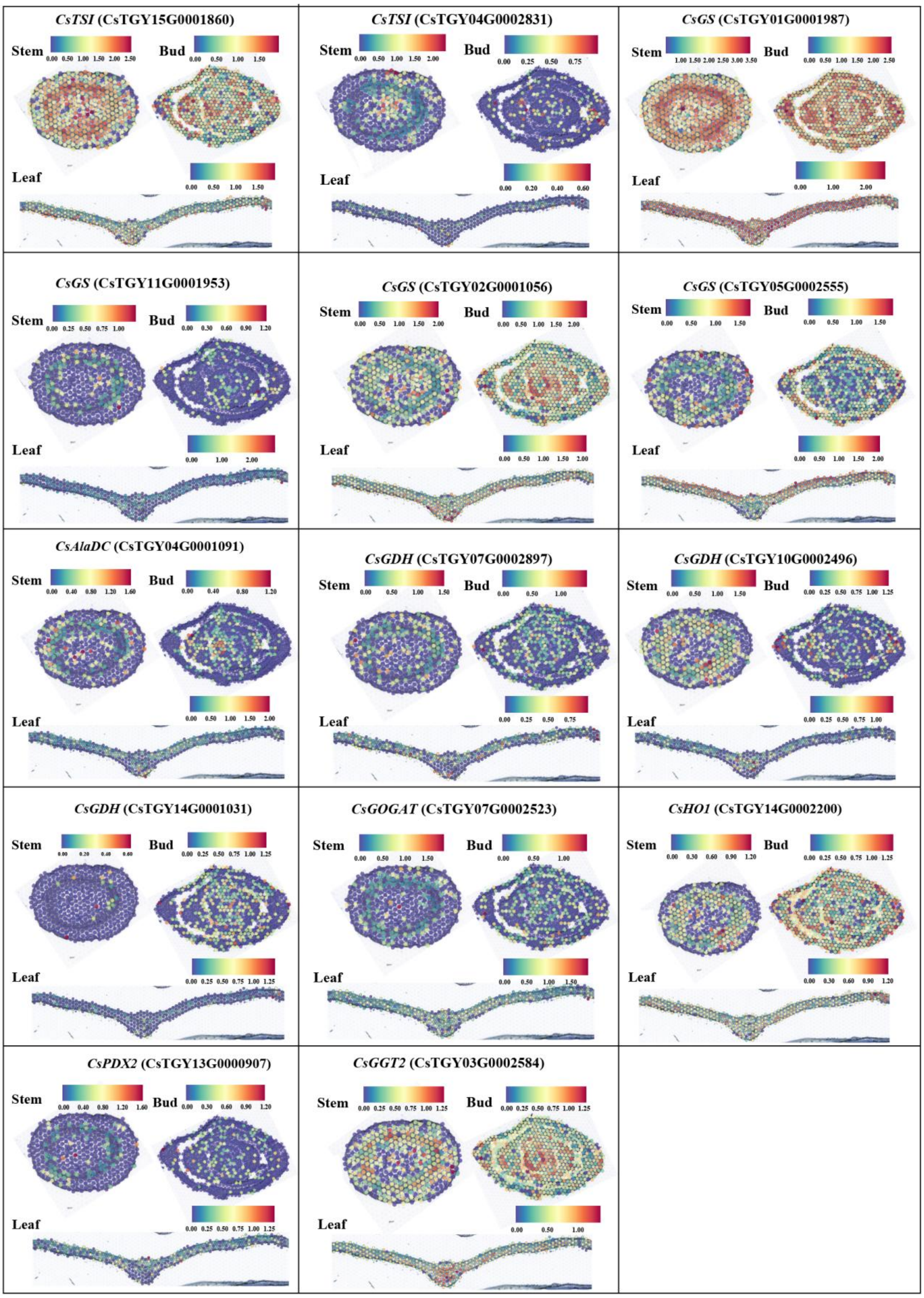

Fig. S8. Spatial expression patterns of structural genes associated with theanine metabolism.

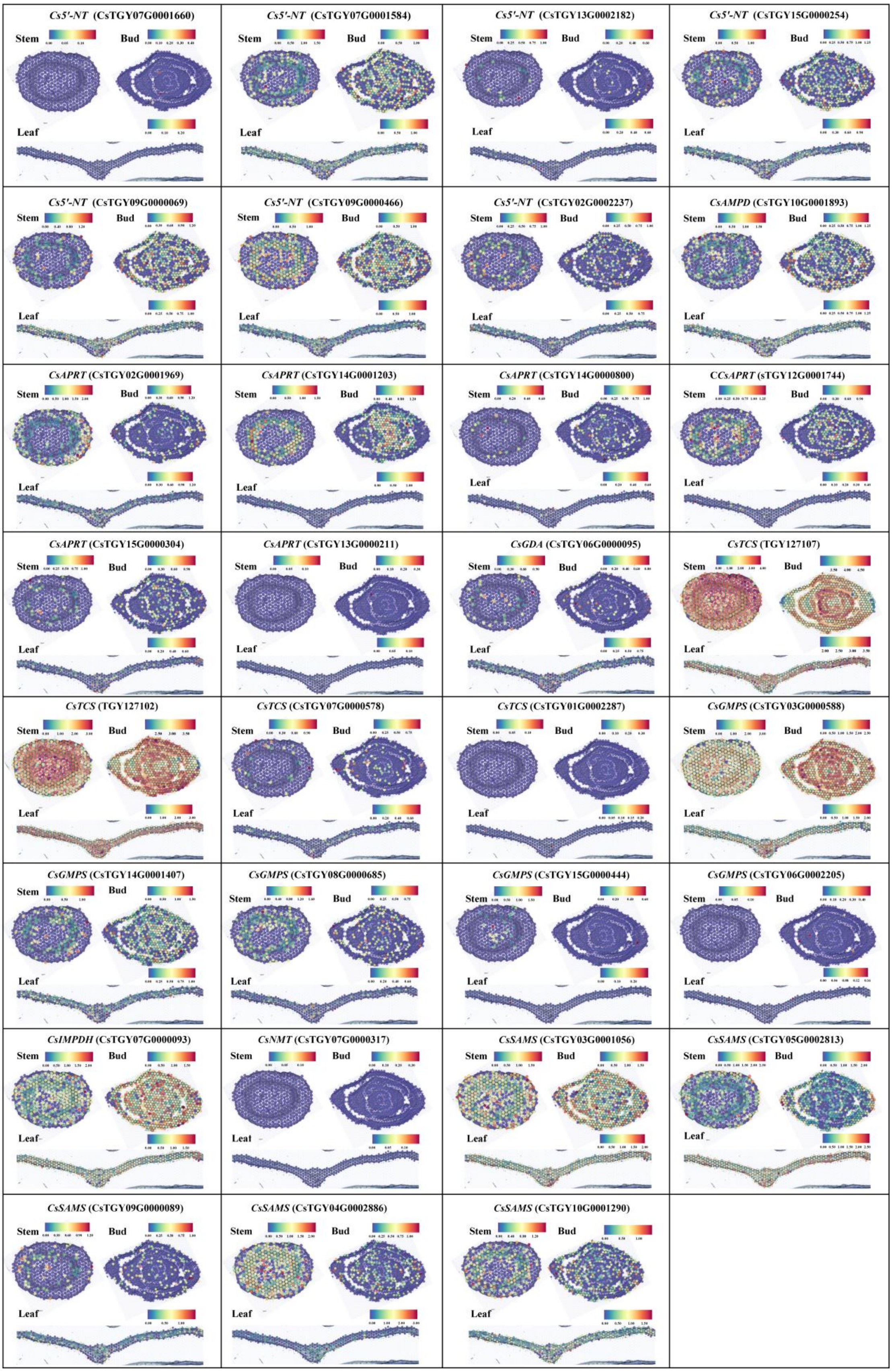

Fig. S9. Spatial expression patterns of structural genes associated with caffeine metabolism.
